# Estimation of life's essential 8 score with incomplete data of individual metrics

**DOI:** 10.3389/fcvm.2023.1216693

**Published:** 2023-07-26

**Authors:** Yi Zheng, Tianyi Huang, Marta Guasch-Ferre, Jaime Hart, Francine Laden, Jorge Chavarro, Eric Rimm, Brent Coull, Hui Hu

**Affiliations:** ^1^ Channing Division of Network Medicine, Department of Medicine, Brigham and Women’s Hospital and Harvard Medical School, Boston, MA, United States; ^2^ Division of Sleep Medicine, Harvard Medical School, Boston, MA, United States; ^3^ Department of Nutrition, Harvard T.H. Chan School of Public Health, Boston, MA, United States; ^4^ Section of Epidemiology, Department of Public Health, University of Copenhagen, Copenhagen, Denmark; ^5^ Center for Basic Metabolic Research, Novo Nordisk Foundation, Copenhagen, Denmark; ^6^ Department of Environmental Health, Harvard T.H. Chan School of Public Health, Boston, MA, United States; ^7^ Department of Epidemiology, Harvard T.H. Chan School of Public Health, Boston, MA, United States; ^8^ Department of Biostatistics, Harvard T.H. Chan School of Public Health, Boston, MA, United States

**Keywords:** cardiovascular health, life’s essential 8, life’s simple 7, machine learning, prediction model

## Abstract

**Background:**

The American Heart Association's Life's Essential 8 (LE8) is an updated construct of cardiovascular health (CVH), including blood pressure, lipids, glucose, body mass index, nicotine exposure, diet, physical activity, and sleep health. It is challenging to simultaneously measure all eight metrics at multiple time points in most research and clinical settings, hindering the use of LE8 to assess individuals' overall CVH trajectories over time.

**Materials and methods:**

We obtained data from 5,588 participants in the Nurses' Health Studies (NHS, NHSII) and Health Professionaĺs Follow-up Study (HPFS), and 27,194 participants in the 2005–2016 National Health and Nutrition Examination Survey (NHANES) with all eight metrics available. Individuals' overall cardiovascular health (CVH) was determined by LE8 score (0–100). CVH-related factors that are routinely collected in many settings (i.e., demographics, BMI, smoking, hypertension, hypercholesterolemia, and diabetes) were included as predictors in the base models of LE8 score, and subsequent models further included less frequently measured factors (i.e., physical activity, diet, blood pressure, and sleep health). Gradient boosting decision trees were trained with hyper-parameters tuned by cross-validations.

**Results:**

The base models trained using NHS, NHSII, and HPFS had validated root mean squared errors (RMSEs) of 8.06 (internal) and 16.72 (external). Models with additional predictors further improved performance. Consistent results were observed in models trained using NHANES. The predicted CVH scores can generate consistent effect estimates in associational studies as the observed CVH scores.

**Conclusions:**

CVH-related factors routinely measured in many settings can be used to accurately estimate individuals' overall CVH when LE8 metrics are incomplete.

## Introduction

Cardiovascular disease (CVD) is the top cause of death both in the United States (US) and globally (1). It is estimated that 80% of CVD is preventable (2). Conventional CVD prevention strategies emphasize the optimizations of classical risk factors such as blood pressure and lipids. However, it is challenging to communicate CVD risk to young individuals with a low absolute 10-year CVD risk. To address this, the American Heart Association (AHA) introduced the Life's Simple 7 (LS7) in 2010, to assess and promote cardiovascular health (CVH) (3), which anchors CVD prevention in health rather than disease to prompt attention to primordial prevention across life course (4). The AHA defined ideal CVH based on seven metrics (LS7), including blood pressure, total cholesterol, glucose, body mass index (BMI), cigarette smoking, diet, and physical activity (3). To better account for factors predictive of CVH, the AHA recently introduced Life's Essential 8 (LE8), an updated construct of CVH with revised quantitative assessment of the 7 existing metrics as well as one new metric focusing on sleep health (5). Previous studies have shown that CVH is not only associated with CVD (6, 7), but also non-CVD outcomes such as cancer (8), cognitive impairment (9), depression (10), and all-cause mortality (11).

In 2016, the AHA announced an ambitious initiative, One Brave Idea (12), with the goal to end coronary heart disease and its consequences. An interim target called “50 × 50 × 50” was proposed in 2018, with the goal of achieving ideal CVH among “≥50% segments of the population ≤50 years old by 2050 or sooner” (13). Previous estimates based on LS7 showed that the prevalence of ideal CVH in the US population is around 50% at 10 years of age and declines to less than 10% by 50 years of age (14, 15). Similarly, recent estimates based on LE8 showed that compared with individuals aged 12–19 years, the mean CVH score is 13.9% lower among those aged 40–64 years (16). Therefore, it is important to understand population-level CVH trajectories and identify factors contributing to different CVH trajectories to promote and preserve CVH. However, to date, population-level CVH estimates are mainly cross-sectional (14, 17–22). Very few studies have examined individuals' CVH trajectories over time (23–28). Among these existing studies, CVH trajectories were determined based on either CVH status sparsely measured over time (e.g., 3 time points in ≥10 years) (23–25), or modified versions of LS7 where not all CVH metrics were considered (25–28). This is mainly due to the challenges of having all CVH metrics simultaneously measured at multiple time points, which substantially hindered the adoption of LE8 to promote and preserve CVH across life course. It remains unclear regarding the performance of a subset of LE8 metrics in estimating overall CVH defined by the full LE8 metrics.

To address this limitation, leveraging data from the Nurses' Health Study (NHS), the Nurses' Health Study II (NHSII), the Health Professional's Follow-up Study (HPFS), and the 2005–2016 National Health and Nutrition Examination Survey (NHANES), we developed and validated models to estimate individuals' overall CVH using CVH-related factors that are routinely collected in many research and clinical settings to enable longitudinal assessment of CVH trajectories even when not all eight CVH metrics are available simultaneously.

## Methods

### Study population

We obtained data from three large nationwide prospective cohorts in the U.S., including NHS and NHSII, with 121,700 and 116,429 female registered nurses recruited in 1976 and 1989, respectively, as well as HPFS, with 51,529 male health professionals recruited in 1986. We also obtained data from the 2005–2016 NHANES, a complex survey with nationally representative samples of noninstitutionalized U.S. adults. A total of 5,588 participants from the cohorts (i.e., 4,114 from NHS, 676 from NHSII, and 798 from HPFS) and 27,194 participants aged 18 and older from the 2005–2016 NHANES with all eight CVH metrics measured.

### Assessment of individual CVH metrics

Blood samples were collected in NHS in 1989–1990 (*n* = 32,826), NHSII in 1996–1999 (*n* = 29,611), and HPFS in 1993–1995 (*n* = 18,159). Among them, a total of 5,030, 785, and 1,388 participants in NHS, NHSII, and HPFS, respectively, had both hemoglobin A1c (HbA1c) and blood lipids measured in the same blood sample. In the 2005–2016 NHANES, HbA1c was measured in whole blood biospecimen using chromatogram, and blood lipids was measured in serum sample using an enzymatic assay (29). Measures of the other six metrics (i.e., BMI, nicotine exposure, blood pressure, diet, physical activity, and sleep health) were obtained in NHS, NHSII, and HPFS based on self-reports from questionnaires closest to blood sample collections (Supplementary Table S1). Previous validation studies showed that these self-reported measures are highly accurate (30–44). Participants in NHS, NHSII, HPFS cohorts were asked about their typical systolic and diastolic blood pressure (i.e., systolic pressure: <105, 105–114, 115–124, 125–134, 135–144, 145–154, 155–164, 165–174, and ≥175 mmHg; diastolic pressure: <65, 65–74, 75–84, 85–89, 90–94, 95–104, and ≥105 mmHg). In NHANES, participants' blood pressures were consecutively measured multiple times with at least 5 min of break between measurements, and the average blood pressure was used. Self-reported history of medications on hypertension (i.e., thiazide diuretics, alpha blockers, beta blockers, calcium channel blockers, angiotensin-converting enzyme inhibitors, Lasix, and other anti-hypertensive medications), diabetes (i.e., insulin, and oral hypoglycemic medications), and hypercholesterolemia (i.e., statin and other cholesterol-lowering medications) was used to determine controlled treatments in both the cohorts and NHANES. BMI was calculated based on self-reported weight and height in the cohorts, while in NHANES, weight and height were measured by physical examinations. Nicotine exposure was assessed by self-reports in both the cohorts and NHANES. In the cohorts, physical activity was computed by summing up the metabolic equivalent of task (MET) hours of each individual activity per week according to corresponding MET score and self-reported hours of the activity (43, 45). In NHANES, physical activity was determined based on self-reported frequency and duration of moderate- and vigorous-intensity leisure time activities, with 4 MET scores assigned to each minute of moderate activities and 8 MET scores assigned to each minute of vigorous activities. Diet was assessed by a >130-item validated food frequency questionnaire in the cohorts (36, 38–42), and by 24-hour dietary recall in NHANES. Sleep health was assessed by self-reported average sleep hours during a 24-hours period in both NHANES and the cohorts.

The eight individual CVH metrics (i.e., blood pressure, lipids, glucose, BMI, nicotine exposure, diet, physical activity, and sleep health) were scored with a range from 0 to 100. Table 1 shows the detailed scoring criteria for each metric. Specifically, we used the same criteria recommended by the AHA to assess blood lipids, nicotine exposure, and physical activity (3, 5). For blood pressure, we used a slightly different sets of cut points because (1) these were the cut-points used in the questionnaires for NHS, NHSII, and HPFS, (2) although the American College of Cardiology (ACC)/AHA hypertension clinical practice guideline set 130/80 mmHg as the cut point for hypertension diagnosis (46), the International Society of Hypertension Global Hypertension Practice Guidelines set average day time ambulatory blood pressures or home blood pressure >135/85 mmHg as the criteria for hypertension diagnosis (47), and (3) it has been shown that any blood pressure over 115/75 increases the risk of CVD (48–50). HbA1c was used to assess the glucose metric since fasting blood glucose was not collected in NHS, NHSII, and HPFS. Moreover, HbA1c test is recommended and clinically used to detect diabetes with high validity and cost-effectiveness (51, 52), and widely used in other studies to assess CVH (53–56). In addition, alternative healthy eating index 2010 (AHEI-2010) was used to measure adherence to a healthy diet pattern based on foods and nutrients that are predictive of chronic disease risk and has been used to assess diet-disease associations in many published studies (57–59). Percentiles of AHEI-2010 scores were used to assess status of diet.

**Table 1:** Scoring criteria of CVH metrics based on life's essential 8.

Metric	Points and criteria
Blood pressure	100: SBP < 115 mmHg and DBP < 75 mmHg 75: SBP 115–124 mmHg and DBP < 75 mmHg 50: SBP 125–134 mmHg or DBP: 75–84 mmHg 25: SBP 135–154 mmHg or DBP 85–94 mmHg 0: SBP ≥ 155 mmHg or DBP ≥ 95 mmHg (Subtract 20 points if treated level)
HbA1c	100: No history of diabetes and HbA1c < 5.7% 60: No diabetes and HbA1c 5.7–6.4% 40: Diabetes with HbA1c < 7.0% 30: Diabetes with HbA1c 7.0–7.9% 20: Diabetes with HbA1c 8.0–8.9% 10: Diabetes with HbA1c 9.0–9.9% 0: Diabetes with HbA1c ≥ 10.0%
Blood lipids	100: Non-HDL cholesterol <130 mg/dl 60: Non-HDL cholesterol 130–159 mg/dl 40: Non-HDL cholesterol 160–189 mg/dl 20: Non-HDL cholesterol 190–219 mg/dl 0: Non-HDL cholesterol ≥220 mg/dl (Subtract 20 points if treated level)
Nicotine exposure	100: Never smoker 75: Former smoker, quit ≥5 year 50: Former smoker, quit 1–5 year 25: Former smoker, quit <1 year 0: Current smoker (Subtract 20 points if living with active indoor smoker in home)
BMI	100: <25 kg/m^2^ 70: 25.0–29.9 kg/m^2^ 0: 30.0–34.9 kg/m^2^ 15: 35.0–39.9 kg/m^2^ 0: ≥40.0 kg/m^2^
Physical activity	100: ≥10.0 MET hours/week 90: 8.0–9.9 MET hours/week 80: 6.0–7.9 MET hours/week 60: 4.0–5.9 MET hours/week 40: 2.0–3.9 MET hours/week 20: 0.1–1.9 MET hours/week 0: 0 MET hours/week
Diet	100: AHEI-2010 score ≥95th percentile 80: AHEI-2010 score between 75th–94th percentile 50: AHEI-2010 score between 50th–74th percentile 25: AHEI-2010 score between 25th–49th percentile 0: AHEI-2010 score <25th percentile
Sleep health	100: 7 ≤ 9 h per night 90: 9 ≤ 10 h per night 70: 6 ≤ 7 h per night 40: 5 ≤ 6 or ≥10 h per night 20: 4 ≤ 5 h per night 0: <4 h per night

AHEI-2010, alternative healthy eating index 2010; BMI, body mass index; CVH, cardiovascular health; DBP, diastolic blood pressure; HbA1c, glycohemoglobin; HDL, high-density lipoprotein; MET, metabolic equivalent of task; SBP, systolic blood pressure.

### Assessment of overall CVH

The outcome in the study is the overall CVH based on all eight LE8 metrics. We generated both a continuous and two binary measures of overall CVH. The continuous overall CVH score was calculated by averaging scores of all eight LE8 metrics (range: 0–100). In addition, we also categorized the continuous CVH score into three categories (i.e., ≥80: high, 50–80: moderate, and <50: low), and two binary outcomes were generated comparing individuals with (1) high CVH vs. moderate or low CVH and (2) low CVH vs. moderate or high CVH.

### Assessment of predictors

Supplementary Figure S1 shows the availabilities of each predictor in NHS, NHSII, and HPFS. We first included predictors that are widely available in NHS, NHSII, and HPFS. These predictors included (1) demographic factors such as age (years), sex (female or male), race/ethnicity (non-Hispanic white, non-Hispanic black, Hispanic, and others), (2) CVH-related factors (measured biennially) such as self-reported hypertension (yes or no), self-reported diabetes (yes or no), and self-reported hypercholesterolmia (yes or no), and (3) CVH metrics (measured biennially) including BMI (both the original BMI value and BMI score defined by LE8) and nicotine exposure (defined by LE8). We further included other CVH metrics that are less frequently collected (i.e., approximately every 4 years) in NHS, NHSII, and HPFS as predictors (Supplementary Figure S1), including self-reported blood pressure, physical activity, diet, and sleep health assessed based on LE8.

### Statistical analyses

**Figure 1:**
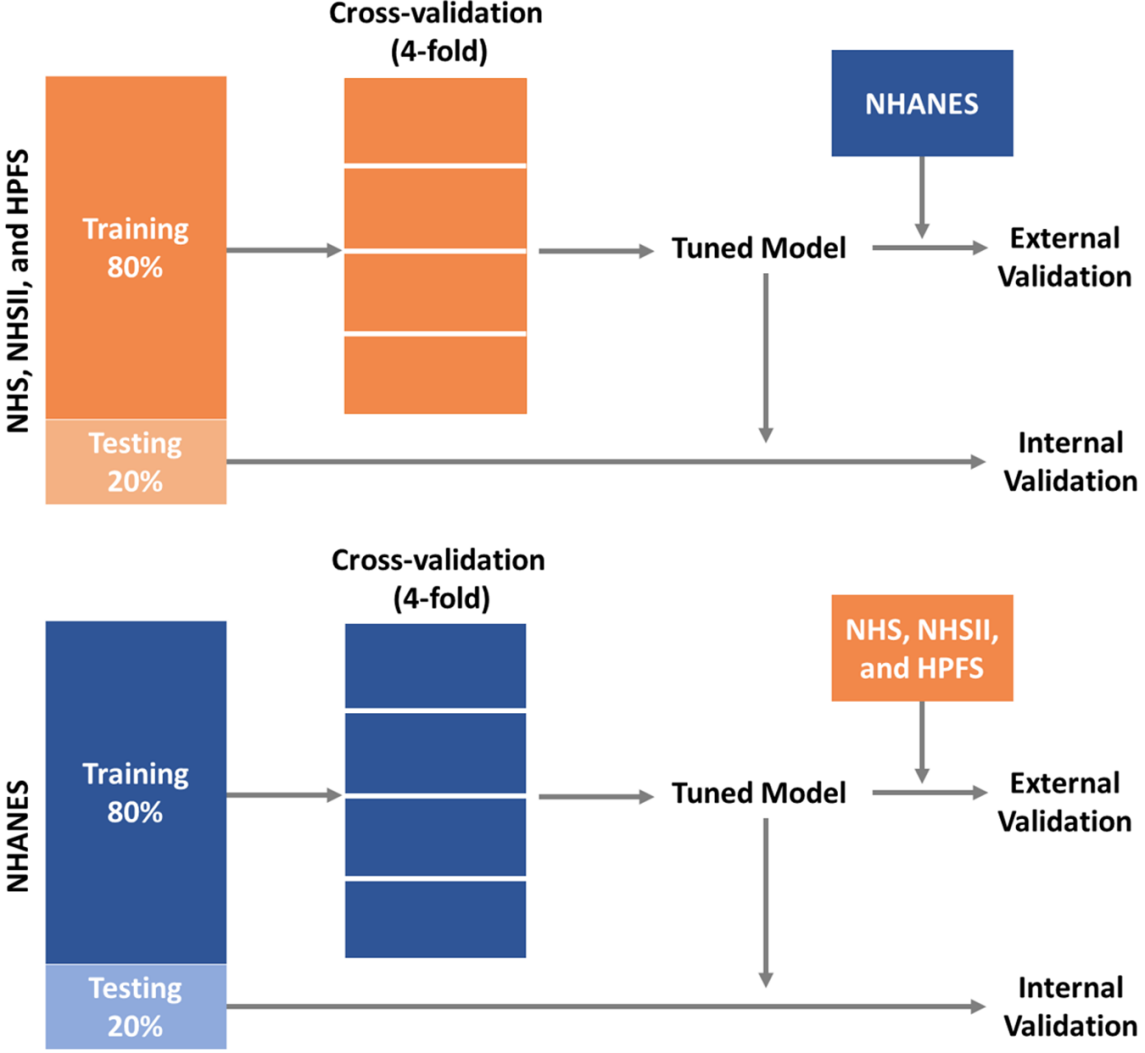
Training and validation pipelines for prediction models of CVH using data from NHS, NHSII, and HPFS, and the 2005–2016 NHANES. CVH, cardiovascular health; HPFS, Health Professional's Follow-up Study; NHANES, the National Health and Nutrition Examination Survey; NHS, Nurses’ Health Study; NHSII, Nurses’ Health Study II.

Descriptive analyses were conducted to examine the distribution of participants' demographics, individual CVH metrics, and overall CVH. Two groups of models were trained separately using data from the cohorts (i.e., NHS, NHSII, and HPFS) and NHANES. Figure 1 shows the model training and testing pipelines. Each group of models contain 16 sets of models each with different predictors: we start by training the base models which included predictors that are routinely collected in NHS, NHSII, and HPFS, such as demographic factors (i.e., age, sex, race/ethnicity), CVH-related factors (i.e., hypertension, hypercholesterolemia, and diabetes), as well as CVH metrics (i.e., BMI and nicotine exposure). We then further included CVH metrics (i.e., blood pressure, physical activity, diet, and sleep health) that are less frequently collected as predictors in additional models (15 sets of models). Of note, percentiles of AHEI-2010 scores were generated separately in the cohorts (i.e., NHS, NHSII, and HPFS) and NHANES for model trainings, and the corresponding cut-points were used to determine diet status in external validations. All models were trained using gradient boosting decision trees implemented by CatBoost (gradient boosting with categorical features support), a highly efficient ensemble-based machine learning model (60). Following the best practice in the field, we randomly split the data into a training set (80%) and a testing set (20%). The training sets were used to tune hyperparameters (i.e., number of iterations, number of trees, learning rate, L2 regularization, tree depth, and border count) using grid searches based on 4-fold cross-validated RMSEs (root mean square errors) for the continuous overall CVH score and AUCs (areas under the receiver operator characteristic curve) for the two binary outcomes (i.e., high CVH vs. moderate/low CVH and low CVH vs. moderate/high CVH). The testing set was then used to perform internal validation. External validations were also conducted using external testing data (e.g., models trained using NHS, NHSII, and HPFS data were externally validated using NHANES data and vice versa). To examine the robustness of model performance in different cohorts, we also generated stratified internal validation results in NHS, NHSII, and HPFS, separately.

To further examine the performance of this approach in real world settings, we conducted sensitivity analyses by assessing whether the predicted CVH scores can generate consistent effect estimates in associational studies. Cox proportional hazards models were used to assess the associations between all-cause mortality and both the observed and predicted LE8 scores in the internal testing sets in NHS, NHSII, and HPFS as well as the NHANES. Hazard ratios (HR) with 95% confidence intervals (CIs) were generated. To account for the complex survey design of the NHANES, a 12-year weight was calculated by dividing the original 2-year weight by 6 for each individual. Models were adjusted for age (continuous), sex (female and male), and race/ethnicity (non-Hispanic White, non-Hispanic Black, Hispanic, and others), and marital status (never married, married or living with partner, and previously married). In addition, in the NHANES, we further adjusted for education (<high school, high school or equivalent, some college, college/graduate or above) and family poverty income ratio (PIR: <1, 1–2, and ≥2).

It has been suggested that the newly introduced LE8 score (0–100 points) is highly correlated with the previous LS7 score (0–14 points) (16). To assess the robustness of our approach, we have conducted sensitivity analyses using CVH measures based on LS7 as the outcomes (Supplementary Table S2). Specifically, the seven individual LS7 metrics (i.e., blood pressure, HbA1c, total cholesterol, smoking, BMI, physical activity, and diet) were categorized into 3 levels: poor (0 point), intermediate (1 point), and ideal (2 points). A continuous CVH score was then calculated by summing up scores of all the seven metrics (range: 0–14). We also generated seven binary measures of overall CVH based on the number of ideal CVH metrics. We used the same modelling pipeline for LS7, with a total of 8 sets of predictors. Similarly, Cox proportional hazards models were also fitted in the internal testing sets to assess the associations between all-cause mortality and both the observed and predicted LS7 scores.

All analyses were conducted using R version 4.1.0 with CatBoost models implemented using the “catboost” R package (61).

## Results

A total of 5,588 and 27,194 participants from the NHS, NHSII, and HPFS cohorts and the 2005–2016 NHANES with complete information on all eight CVH metrics were included in this study, respectively. Table 2 shows the distributions of participants' demographic characteristics, medical history, overall LE8 score, and individual LE8 metric scores. Compared with participants in the NHANES, participants in NHS, NHSII, and HPFS were older, more likely to be non-Hispanic White, less likely to have hypertension, diabetes, and hypercholesterolemia, and more likely to have better overall CVH. In addition, participants in NHS, NHSII, and HPFS were also more likely to have more optimal individual CVH metrics including BMI, nicotine exposure, physical activity, diet, and sleep health, while those in the NHANES were more likely to have better status in blood pressure, HbA1c, and blood lipids (all *p* < 0.001).

**Table 2:** Characteristics of participants in NHS, NHSII, and HPFS, and the 2005–2016 NHANES included in developing prediction models of life's essential 8 score.

Characteristics	NHS, NHSII, and HPFS cohorts				NHANES
	NHS	NHSII	HPFS	Total	
	(*n* = 4,114)	(*n* = 676)	(*n* = 798)	(*n* = 5,588)	(*n* = 27,194)
	Mean ± SD/*n* (%)				
Age (years)	59.6 ± 6.5	45.2 ± 4.1	62.9 ± 8.7	58.3 ± 8.3	48.8 ± 17.8
BMI (kg/m^2^)	26.1 ± 5.1	26.0 ± 5.7	25.9 ± 3.3	26.1 ± 5.0	29.0 ± 6.8
Sex					
Male	0 (0.0)	0 (0.0)	798 (100.0)	798 (14.3)	13,219 (48.6)
Female	4,114 (100.0)	676 (100.0)	0 (0.0)	4,790 (85.7)	13,975 (51.4)
Race/ethnicity					
Non-hispanic white	3,872 (94.1)	656 (97.0)	426 (53.4)	4,954 (88.7)	12,180 (44.8)
Non-hispanic black	19 (0.5)	5 (0.7)	0 (0.0)	24 (0.4)	5,471 (20.1)
Hispanic	30 (0.7)	7 (1.0)	4 (0.5)	41 (0.7)	7,059 (26.0)
Others	193 (4.7)	8 (1.2)	368 (46.1)	569 (10.2)	2,484 (9.1)
Hypertension					
No	3,041 (73.9)	605 (89.5)	602 (75.4)	4,248 (76.0)	17,721 (65.2)
Yes	1,073 (26.1)	71 (10.5)	196 (24.6)	1,340 (24.0)	9,437 (34.7)
Missing	0 (0)	0 (0)	0 (0)	0 (0)	36 (0.1)
Diabetes					
No	3,574 (86.9)	658 (97.3)	760 (95.2)	4,992 (89.3)	23,216 (85.4)
Yes	540 (13.1)	18 (2.7)	38 (4.8)	596 (10.7)	3,978 (14.6)
Hypercholesterolemia					
No	2,536 (61.6)	577 (85.4)	570 (71.4)	3,683 (65.9)	14,010 (51.5)
Yes	1,578 (38.4)	99 (14.6)	228 (28.6)	1,905 (34.1)	8,874 (32.6)
Missing	0 (0)	0 (0)	0 (0)	0 (0)	4,310 (15.8)
Overall CVH					
LE8 score (0–100)	65.4 ± 13.1	73.8 ± 14.2	60.0 ± 10.3	65.6 ± 13.4	61.6 ± 14.3
Categorical measure					
Low (LE8 score < 50)	503 (12.2)	37 (5.5)	134 (16.8)	674 (12.1)	5,743 (21.1)
Moderate (LE8 score 50–80)	3,040 (73.9)	379 (56.1)	653 (81.8)	4,072 (72.9)	18,424 (67.8)
High (LE8 score ≥80)	571 (13.9)	260 (38.5)	11 (1.4)	842 (15.1)	3,027 (11.1)
Individual LE8 metric scores					
Blood pressure	45.9 ± 28.9	65.1 ± 29.8	45.3 ± 24.4	48.1 ± 29.1	59.6 ± 31.8
HbA1c	80.9 ± 28.0	95.7 ± 15.8	1.5 ± 0.6	71.4 ± 38.0	80.4 ± 27.1
Blood lipids	42.1 ± 33.3	63.1 ± 34.3	53.7 ± 31.8	46.3 ± 34.0	64.4 ± 31.0
Nicotine exposure	71.0 ± 35.3	81.2 ± 32.0	79.7 ± 26.5	73.4 ± 34.0	70.5 ± 39.5
BMI	75.8 ± 29.1	77.3 ± 30.1	78.2 ± 22.5	76.3 ± 28.4	60.5 ± 33.5
Physical activity	79.5 ± 28.9	80.7 ± 29.6	89.6 ± 23.8	81.1 ± 28.5	44.7 ± 46.7
Diet	39.4 ± 30.8	39.0 ± 32.5	42.0 ± 33.1	39.8 ± 31.3	31.9 ± 26.6
Sleep health	88.5 ± 19.5	88.5 ± 21.8	89.7 ± 20.2	88.6 ± 19.9	80.8 ± 25.8

BMI, body mass index; CVH, cardiovascular health; HbA1c, glycohemoglobin; LE8, life's essential 8.

**Figure 2:**
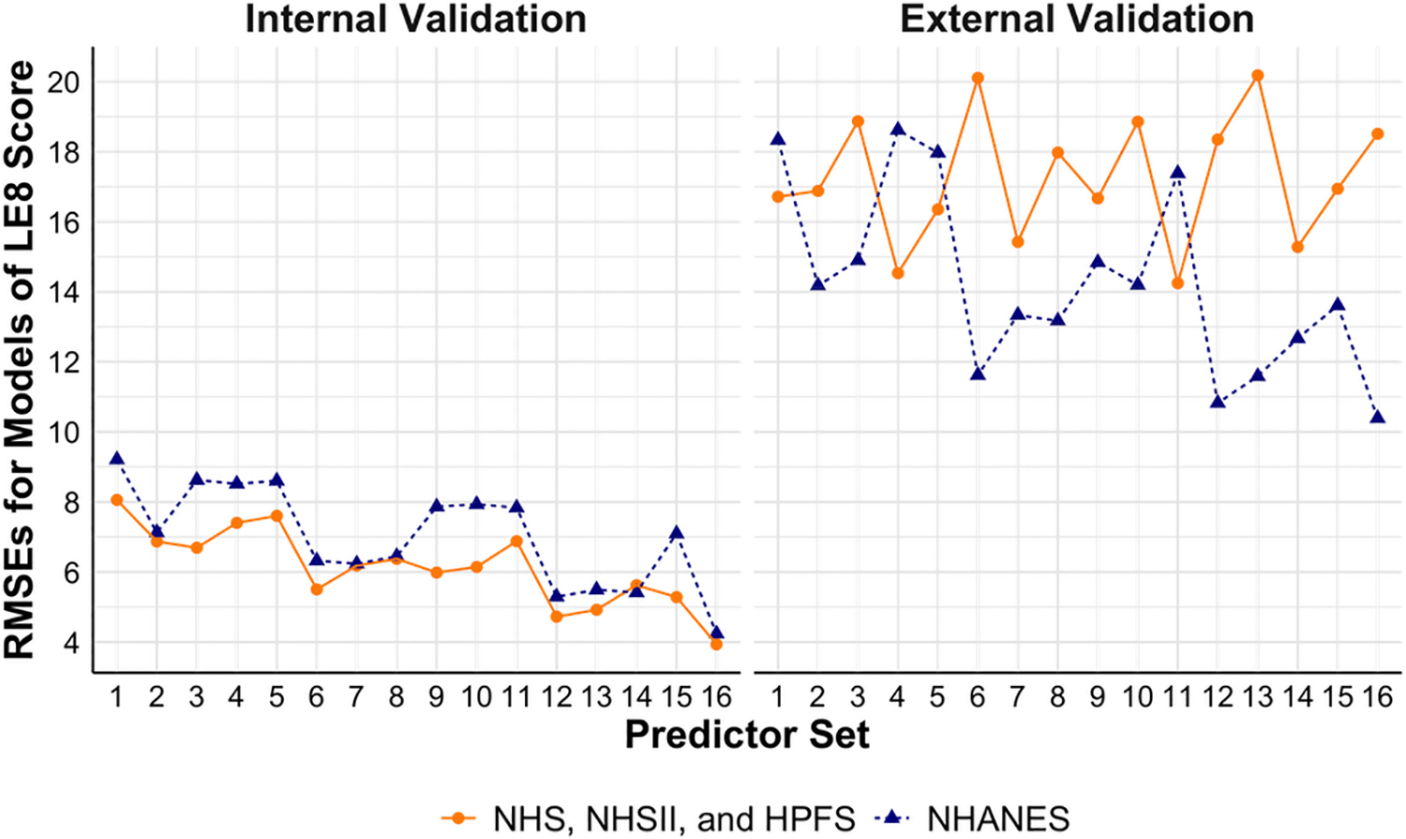
Performance of models to estimate continuous LE8 score using NHS, NHSII, and HPFS (*n* = 5,588), and NHANES (*n* = 27,194). Set 1 (i.e., base model): age, sex, race/ethnicity, BMI, smoking, hypertension, hypercholesterolemia, and diabetes; Set 2: +physical activity; Set 3: +diet; Set 4: +blood pressure; Set 5: +sleep health; Set 6: +physical activity + diet; Set 7: +physical activity + blood pressure; Set 8: +physical activity + sleep health; Set 9: +diet + blood pressure; Set 10: +diet + sleep health; Set 11: +blood pressure + sleep health; Set 12: +physical activity + diet + blood pressure; Set 13: +physical activity + diet + sleep health; Set 14: +physical activity + blood pressure + sleep health; Set 15: +diet + blood pressure + sleep health; Set 16: +physical activity + diet + blood pressure + sleep health. BMI, body mass index; CVH, cardiovascular health; HPFS, Health Professional's Follow-up Study; LE8, life's essential 8; NHANES, the National Health and Nutrition Examination Survey; NHS, Nurses’ Health Study; NHSII, Nurses’ Health Study II; RMSE, root mean square error.

Hyperparameters tuned based on grid searches are presented in Supplementary Tables S3, S4 for the models trained using the cohorts and NHANES, respectively. Figure 2 shows the performance of models to estimate the continuous overall CVH score based on LE8. Internally and externally validated RMSEs of 8.06 and 16.72 were observed, respectively, in base models trained using the cohorts. Similarly, in base models trained using NHANES, internally and externally validated RMSEs of 9.21 and 18.33 were observed. Models additionally including physical activity, diet, blood pressure, and sleep health had the best internally validated RMSEs (3.94 in the best model trained using the cohorts, and 4.24 in the best model trained using NHANES). Models trained using the cohorts with additional predictors including blood pressure and sleep health had the best externally validated RMSE of 14.25, while models trained using NHANES had best externally validated RMSE of 10.39 with additional predictors including physical activity, diet, blood pressure, and sleep health.

**Figure 3:**
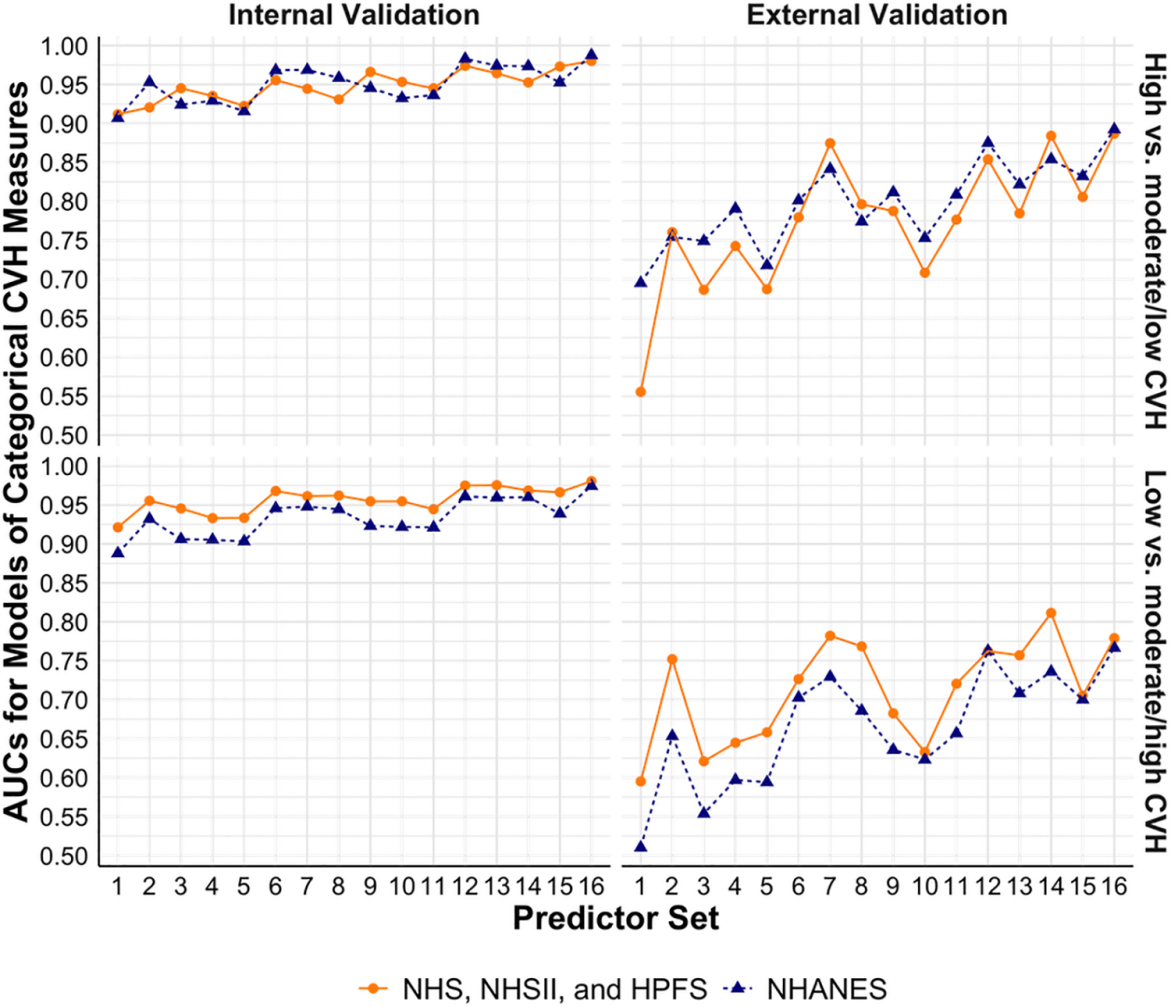
Performance of models to estimate categorical CVH measures based on LE8 score using NHS, NHSII, and HPFS (*n* = 5,588), and NHANES (*n* = 27,194). Set 1 (i.e., base model): age, sex, race/ethnicity, BMI, smoking, hypertension, hypercholesterolemia, and diabetes; Set 2: +physical activity; Set 3: +diet; Set 4: +blood pressure; Set 5: +sleep health; Set 6: +physical activity + diet; Set 7: +physical activity + blood pressure; Set 8: +physical activity + sleep health; Set 9: +diet + blood pressure; Set 10: +diet + sleep health; Set 11: +blood pressure + sleep health; Set 12: +physical activity + diet + blood pressure; Set 13: +physical activity + diet + sleep health; Set 14: +physical activity + blood pressure + sleep health; Set 15: +diet + blood pressure + sleep health; Set 16: +physical activity + diet + blood pressure + sleep health. AUC, area under the receiver operator characteristic curve; BMI, body mass index; CVH, cardiovascular health; HPFS, Health Professional's Follow-up Study; LE8, Life's Essential 8; NHANES, the National Health and Nutrition Examination Survey; NHS, Nurses’ Health Study; NHSII, Nurses’ Health Study II.

Figure 3 shows the performance of models to estimate binary CVH outcomes. In models trained using the cohorts, the base models had validated AUCs of 0.91 and 0.92 (internal) and 0.56 and 0.60 (external) for high vs. moderate/low CVH and low vs. moderate/high CVH, respectively. Similarly, the base models trained using NHANES had internally validated AUCs of 0.91 and 0.89 and externally validated AUCs of 0.70 and 0.51 for the two binary CVH outcomes, respectively. Models with additional predictors such as physical activity, diet, blood pressure, and sleep health had better performance, with the best validated AUCs of 0.98 and 0.98 (internal) and 0.89 and 0.78 (external) in models trained using the cohorts, and 0.99 and 0.97 (internal) and 0.89 and 0.77 (external) in models trained using NHANES for the two binary CVH outcomes, respectively.

Supplementary Tables S5, S6 show the detailed results for each model. Consistent results were observed in internal validations by cohort (Supplementary Table S7).

**Figure 4:**
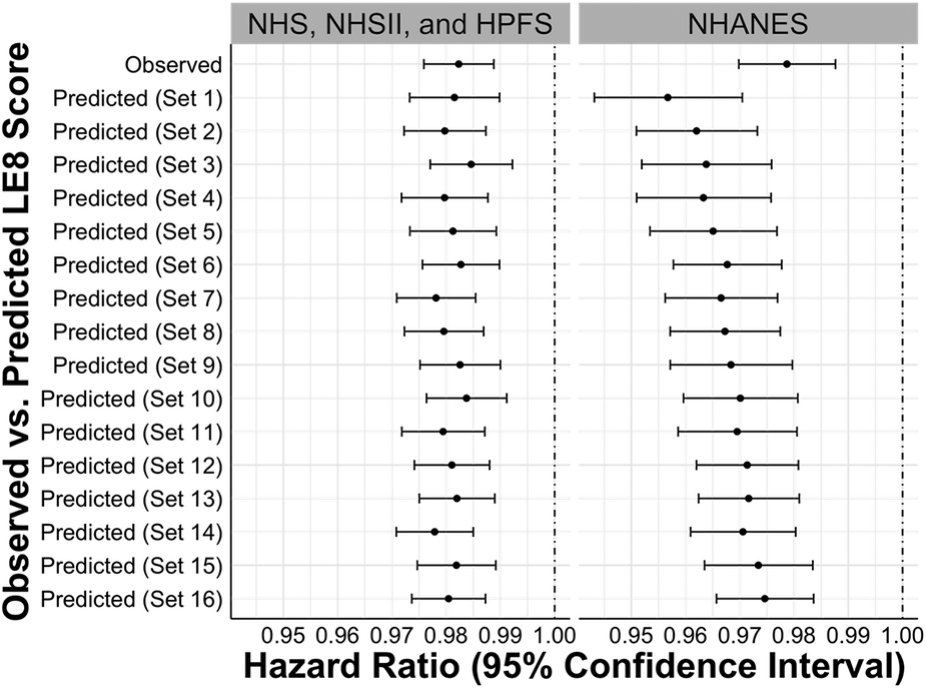
Hazard ratios and 95% confidence intervals for the associations between observed vs. predicted LE8 scores and all-cause mortality in internal testing sets of NHS, NHSII, and HPFS (*n* = 5,588), and NHANES (*n* = 27,194). Set 1 (i.e., base model): age, sex, race/ethnicity, BMI, smoking, hypertension, hypercholesterolemia, and diabetes; Set 2: +physical activity; Set 3: +diet; Set 4: +blood pressure; Set 5: +sleep health; Set 6: +physical activity + diet; Set 7: +physical activity + blood pressure; Set 8: +physical activity + sleep health; Set 9: +diet + blood pressure; Set 10: +diet + sleep health; Set 11: +blood pressure + sleep health; Set 12: +physical activity + diet + blood pressure; Set 13: +physical activity + diet + sleep health; Set 14: +physical activity + blood pressure + sleep health; Set 15: +diet + blood pressure + sleep health; Set 16: +physical activity + diet + blood pressure + sleep health. BMI, body mass index; CVH, cardiovascular health; HPFS, Health Professional's Follow-up Study; LE8, life's essential 8; NHANES, the National Health and Nutrition Examination Survey; NHS, Nurses’ Health Study; NHSII, Nurses’ Health Study II.

Figure 4 presents the HRs and 95% CIs for all-cause mortality. In the cohorts, one unit increase in the observed LE8 score was associated with significantly lower hazards of all-cause mortality (HR: 0.982, 95% CI: 0.976–0.989). Consistent results were observed in models using predicted LE8 scores based on different sets of predictors. Similarly, in the NHANES, no statistically significant difference was found between the associations of all-cause mortality with the observed and predicted LE8 scores.

To further assess the robustness of our approach, we conducted sensitivity analyses using CVH measures based on LS7 (Supplementary Table S2). Supplementary Table S8 shows the distributions of demographic characteristics, medical history, overall LS7 CVH, and individual LS7 metrics. Data from 1999 to 2004 NHANES were not used in the main analyses based on LE8 since sleep health was not available, however, they were included in the sensitivity analyses. A total of 8,500 and 39,933 participants from the cohorts and the 1999–2016 NHANES with complete information on all seven LS7 metrics were included in this study, respectively. Consistent with findings for LE8, participants in the cohorts were less likely to have hypertension, diabetes, and hypercholesterolemia and had better overall CVH, compared with participants in the NHANES. Participants in the cohorts were also more likely to have ideal status for individual CVH metrics including BMI, cigarette smoking, physical activity, and diet, while those in the NHANES were more likely to have ideal blood pressure and total cholesterol (all *p* < 0.001).

Supplementary Tables S9, S10 show tuned hyperparameters for the models trained using the cohorts and NHANES, respectively. Supplementary Figures S2, S3 show the performance of models for the continuous CVH score and binary overall CVH measures assessed by LS7. In base models trained using the cohorts, validated RMSEs of 1.47 (internal) and 2.37 (external) and validated AUCs ranging from 0.85 to 0.98 (internal) and 0.74 to 0.90 (external) were observed. Similarly, the base models trained using NHANES had validated RMSEs of 1.55 (internal) and 3.19 (external) and validated AUCs ranging from 0.85 to 0.97 (internal) and 0.77 to 0.87 (external). Models with additional predictors such as physical activity, diet, and/or blood pressure had better performance, with the best validated RMSEs of 0.86 (internal) and 1.81 (external) and validated AUCs ranging from 0.96 to 0.99 (internal) and 0.79 to 0.94 (external) in models trained using the cohorts, and the best validated RMSEs of 0.82 (internal) and 1.92 (external) and validated AUCs ranging from 0.95 to 0.99 (internal) and 0.89 to 0.98 (external) in models trained using NHANES. Supplementary Tables S11, S12 shows the detailed results for each model. Results of stratified internal validations for models of LS7 in each of the cohorts are shown in Supplementary Table S13.

Supplementary Figure S4 presents associations between all-cause mortality and the observed and predicted LS7 scores. Similar to the results observed for the LE8 scores, no statistically significant difference was observed in the associations based on the observed vs. predicted LE7 scores.

## Discussion

Leveraging data from three nationwide prospective cohorts (i.e., NHS, NHSII, and HPFS) and a series of cross-sectional nationally representative data from the NHANES, we developed and validated several sets of models to estimate individuals' overall CVH status defined by LE8 when not all eight metrics are available. We found that information routinely collected and widely available in many research studies and clinical settings (e.g., age, sex, race/ethnicity, BMI, nicotine exposure, hypertension, hypercholesterolemia, and diabetes) can be used to accurately estimate individuals' overall CVH status. Consistent results were observed in sensitivity analyses defining CVH outcomes based on LS7. In addition, the predicted CVH scores can generate consistent effect estimates in associational studies as the observed CVH scores.

Both the original LS7 and the recently updated LE8 metrics introduced by the AHA emphasize primordial prevention, and have great potential to guide and improve CVD prevention (3, 5). It has been shown that individuals' overall CVH declines with age (14–16). A recent pooled cohort analysis on trajectories of clinical CVH scores (based on BMI, blood pressure, cholesterol, and blood glucose) identified two inflection points in late adolescence (i.e., 16.9 years) and early middle age (i.e., 37.2 years) during which the decline of CVH accelerates (28). It is thus important to identify and understand factors contributing to CVH declines at different stages of life. However, due to the challenges to simultaneously measure all eight LE8 (or seven LS7) CVH metrics over time, most existing studies on CVH are cross-sectional (14, 17–22), and the few longitudinal studies which examined individuals' CVH trajectories over time either only had CVH sparsely measured over time (e.g., ≤3 time points in ≥10 years) or used modified versions of LE8 or LS7 (e.g., the clinical CVH score) (23–28). The models developed and validated in this study provide a cost-effective and feasible solution to enable longitudinal assessment of CVH trajectories in multiple settings when not all eight LE8 (or seven LS7) CVH metrics are available.

In this study, we observed great model performance in internal validations for different predictors-outcome pairs in models either trained using the cohorts (i.e., NHS, NHSII, and HPFS) or the NHANES. This is not unexpected as many of the CVH metrics included in LE8 and LS7 are highly correlated, and therefore, it is plausible to use some but not all eight LE8 (or seven LS7) metrics along with other CVH-related factors to estimate individuals' overall CVH. This is supported by results from a recent study, which used 13-year electronic health records with measures of five CVH metrics (i.e., smoking, BMI, blood pressure, glucose, and cholesterol) and found that future individual CVH metrics can be reliably predicted using previous measures of these metrics (27). In addition, we also showed that the predicted CVH scores can generate consistent effect estimates in associational studies as the observed CVH scores. Our findings suggest that in research and clinical settings without all eight LE8 (or seven LS7) CVH metrics measured at every time point, using the few CVH metrics and related factors routinely collected can accurately estimate individuals' overall CVH, making it feasible to examine trajectories of overall CVH over time.

Compared with the results from internal validations, the models performed relatively worse in external validations, which may be mainly caused by differences between the data used in internal and external validations, including (1) different study populations (e.g., NHS, NHSII, and HPFS included only health professions and participants are older, while the NHANES included the general population), and (2) different measurement methods of individual CVH metrics and predictors (e.g., blood pressures were based on self-report in NHS, NHSII, and HPFS, while the NHANES used the average blood pressure from consecutive measurements). These results suggest that while directly using off-the-shelf models pretrained using other data sources (e.g., NHANES) are feasible, when possible, it is ideal to retrain and validate models for specific research or clinical settings, especially when the targeted populations or measurement methods are different from the original data source used to develop the pretrained models.

There are several strengths and some limitations to note. This is the first effort to estimate individuals' overall CVH when not all eight LE8 (or seven LS7) CVH metrics are available. We showed that the few CVH metrics and related factors routinely collected in many research and clinical settings can be used to accurately estimate individuals' overall CVH. This is especially valuable to longitudinal studies focusing on CVH trajectories as it enables inclusions of data from more time points to better characterize longitudinal changes in overall CVH. It is also clinically relevant by providing a cost-effective and feasible way to track individuals' CVH over time. In addition, using three large nationwide prospective cohorts (NHS, NHSII, and HPFS) and the nationally representative NHANES, the results observed, and implications drawn from this study are generalizable to other populations and study settings. One limitation to note is the relatively worse model performance in external validations, which suggested that directly applying off-the-shelf models pretrained using data from other population or setting may yield less accurate estimations. However, the consistently great model performance observed in internal validations using both the cohorts (i.e., NHS, NHSII, and HPFS) and NHANES data provide strong evidence suggesting that individuals’ overall CVH can be accurately estimated with retrained and fine-tuned models for specific research or clinical settings.

## Conclusions

Using data from three large nationwide prospective cohorts (i.e., NHS, NHSII, and HPFS) and a nationally representative survey (i.e., NHANES), we showed that CVH-related factors routinely measured in many research and clinical settings can be used to accurately estimate individuals' overall CVH even when not all eight LE8 (or seven LS7) metrics are available. In summary, the approach introduced in this study provides a cost-effective and feasible way to estimate individuals' overall CVH in multiple settings and is especially valuable to characterize individuals' CVH trajectories over time.

## Data Availability

Publicly available datasets were analyzed in this study. This data can be found here: Nurses’ Health Studies: https://nurseshealthstudy.org/ Health Professionals Follow-Up Study: https://www.hsph.harvard.edu/hpfs/ NHANES: https://www.cdc.gov/nchs/nhanes/index.htm.
