# Supplementary material for: Estimation of life's essential 8 score with incomplete data of individual metrics
Source: Front Cardiovasc Med. 2023 Jul 26;10:1216693. doi: 10.3389/fcvm.2023.1216693 (PMC10410141; doi:10.3389/fcvm.2023.1216693)
Supplement: Supplementary file 1 [file Table1.docx]

Supplementary Material

**Estimation of Life’s Essential 8 Score with Incomplete Data of Individual Metrics**

Yi Zheng^1^, Tianyi Huang^1,2^, Marta Guasch-Ferre^3,4,5^, Jaime Hart^1,6^, Francine Laden^1,6,7^, Jorge Chavarro^1,3,7^, Eric Rimm^1,3,7^, Brent Coull^6,8^, Hui Hu^1*^

^1^ Channing Division of Network Medicine, Department of Medicine, Brigham and Women’s Hospital and Harvard Medical School, Boston, Massachusetts, USA;

^2^ Division of Sleep Medicine, Harvard Medical School, Boston, Massachusetts, USA;

^3^ Department of Nutrition, Harvard T.H. Chan School of Public Health, Boston, Massachusetts, USA;

^4^ Section of Epidemiology, Department of Public Health, University of Copenhagen, Copenhagen, Denmark;

^5^ Novo Nordisk Foundation Center for Basic Metabolic Research, Copenhagen, Denmark;

^6^ Department of Environmental Health, Harvard T.H. Chan School of Public Health, Boston, Massachusetts, USA;

^7^ Department of Epidemiology, Harvard T.H. Chan School of Public Health, Boston, Massachusetts, USA;

^8^ Department of Biostatistics, Harvard T.H. Chan School of Public Health, Boston, Massachusetts, USA;

***Correspondence:**

Hui Hu^*^

hui.hu@channing.harvard.edu

**Supplemental Table 1.** Timing of blood sample collections and questionnaires used to assess CVH metrics in NHS, NHSII, and HPFS.

| **Cohort** | **Blood Sample**  **(Year of Collection)** | **Questionnaire from Closest Follow-up Cycles with Available Data (Year of Collection)** | | | | | | |
| --- | --- | --- | --- | --- | --- | --- | --- | --- |
|  |  | **Blood Pressure** | **BMI** | **Cigarette Smoking** | **Physical  Activity** | **Diet** | **Sleep** | **Medications** |
| NHS | 1989-1991 | 1990 | 1990 | 1990 | 1992 | 1990 | 1986 | 1988 |
| NHSII | 1996-1999 | 1999 | 1999 | 1999 | 2001 | 1999 | 2001 | 2001 |
| HPFS | 1993-1995 | 1992, 1996 | 1994, 1996 | 1994, 1996 | 1994, 1996 | 1994 | 2000 | 1994, 1996 |
| Abbreviations: BMI, body mass index; CVH, cardiovascular health; HPFS, Health Professionals Follow-up Study; NHS, Nurses' Health Study; NHSII, Nurses' Health Study II. | | | | | | | | |

**Supplemental Table 2.** Definitions of poor, intermediate, and ideal CVH metrics based on Life’s Simple 7.

| **CVH metrics** | **Poor** | **Intermediate** | **Ideal** |
| --- | --- | --- | --- |
| Blood pressure | SBP≥135mmHg or DBP≥85mmHg | SBP 115-134mmHg, or DBP 75-84mmHg, or treated to goal | SBP<115mmHg and DBP<75mmHg, untreated |
|  |  |  |  |
| HbA1c | >6.4% | 5.7-6.4% or treated to goal | <5.7%, untreated |
|  |  |  |  |
| Total cholesterol | ≥240mg/dL | 200-239mg/dL or treated to goal | <200mg/dL, untreated |
|  |  |  |  |
| Smoking | Current smoking | Former, quit ≤12 months previously | Never or quit >12 months previously |
|  |  |  |  |
| BMI | ≥30.0kg/m^2^ | 25.0-29.9kg/m^2^ | <25.0kg/m^2^ |
|  |  |  |  |
| Physical activity | None | <10 MET hours/week | ≥10 MET hours/week |
|  |  |  |  |
| Diet | AHEI-2010 Tertile 1 | AHEI-2010 Tertile 2 | AHEI-2010 Tertile 3 |
| Abbreviations: AHEI-2010, alternative healthy eating index 2010; BMI, body mass index; CVH, cardiovascular health; DBP, diastolic blood pressure; HbA1c, glycohemoglobin; MET, metabolic equivalent of task; SBP, systolic blood pressure. | | | |

**Supplemental Table 3.** Optimal hyperparameters of predictive models of CVH based on LE8 tuned by cross-validation in the training set using NHS, NHSII, and HPFS (n=5,588).

| Outcomes and Predictors^a^ | Number of Iteration | Learning rate | Tree depth | Border count | L2 regularization |
| --- | --- | --- | --- | --- | --- |
| LE8 score |  |  |  |  |  |
| Predictor Set 1 | 2047 | 0.005 | 6 | 16 | 5 |
| Predictor Set 2 | 1183 | 0.01 | 6 | 32 | 5 |
| Predictor Set 3 | 1254 | 0.01 | 6 | 32 | 5 |
| Predictor Set 4 | 1850 | 0.005 | 6 | 32 | 0.5 |
| Predictor Set 5 | 2085 | 0.005 | 6 | 64 | 5 |
| Predictor Set 6 | 2407 | 0.005 | 6 | 32 | 1 |
| Predictor Set 7 | 1132 | 0.01 | 6 | 32 | 0.1 |
| Predictor Set 8 | 2452 | 0.005 | 6 | 16 | 0.1 |
| Predictor Set 9 | 1088 | 0.01 | 6 | 64 | 0.5 |
| Predictor Set 10 | 2295 | 0.005 | 6 | 64 | 5 |
| Predictor Set 11 | 895 | 0.01 | 6 | 64 | 0.1 |
| Predictor Set 12 | 2613 | 0.005 | 6 | 64 | 0.1 |
| Predictor Set 13 | 1146 | 0.01 | 6 | 32 | 1 |
| Predictor Set 14 | 1102 | 0.01 | 6 | 32 | 0.1 |
| Predictor Set 15 | 1169 | 0.01 | 6 | 64 | 0.5 |
| Predictor Set 16 | 2740 | 0.005 | 6 | 64 | 0.1 |
| High vs. Moderate/Low CVH | | | | | |
| Predictor Set 1 | 126 | 0.05 | 8 | 64 | 0.1 |
| Predictor Set 2 | 916 | 0.01 | 7 | 32 | 0.1 |
| Predictor Set 3 | 957 | 0.01 | 6 | 16 | 0.1 |
| Predictor Set 4 | 138 | 0.05 | 7 | 32 | 0.1 |
| Predictor Set 5 | 125 | 0.05 | 6 | 32 | 0.1 |
| Predictor Set 6 | 1381 | 0.01 | 6 | 32 | 0.1 |
| Predictor Set 7 | 966 | 0.01 | 6 | 64 | 0.1 |
| Predictor Set 8 | 177 | 0.05 | 6 | 32 | 0.1 |
| Predictor Set 9 | 201 | 0.05 | 6 | 16 | 0.5 |
| Predictor Set 10 | 1184 | 0.01 | 6 | 64 | 0.1 |
| Predictor Set 11 | 186 | 0.05 | 6 | 32 | 0.5 |
| Predictor Set 12 | 1094 | 0.01 | 6 | 64 | 0.1 |
| Predictor Set 13 | 190 | 0.05 | 6 | 64 | 0.1 |
| Predictor Set 14 | 1222 | 0.01 | 6 | 64 | 0.1 |
| Predictor Set 15 | 2000 | 0.005 | 6 | 16 | 0.1 |
| Predictor Set 16 | 179 | 0.05 | 6 | 16 | 0.1 |
| Low vs. Moderate/High CVH | | | | | |
| Predictor Set 1 | 86 | 0.05 | 6 | 16 | 0.5 |
| Predictor Set 2 | 164 | 0.05 | 6 | 16 | 1 |
| Predictor Set 3 | 141 | 0.05 | 6 | 16 | 1 |
| Predictor Set 4 | 85 | 0.05 | 6 | 64 | 1 |
| Predictor Set 5 | 1354 | 0.01 | 6 | 64 | 5 |
| Predictor Set 6 | 139 | 0.05 | 6 | 64 | 0.1 |
| Predictor Set 7 | 143 | 0.05 | 6 | 16 | 1 |
| Predictor Set 8 | 305 | 0.05 | 6 | 16 | 5 |
| Predictor Set 9 | 92 | 0.05 | 6 | 16 | 0.1 |
| Predictor Set 10 | 1105 | 0.01 | 6 | 64 | 5 |
| Predictor Set 11 | 197 | 0.05 | 6 | 16 | 5 |
| Predictor Set 12 | 784 | 0.01 | 6 | 64 | 0.5 |
| Predictor Set 13 | 1447 | 0.01 | 6 | 16 | 1 |
| Predictor Set 14 | 164 | 0.05 | 6 | 64 | 1 |
| Predictor Set 15 | 231 | 0.05 | 6 | 32 | 5 |
| Predictor Set 16 | 1322 | 0.01 | 6 | 64 | 1 |
| Abbreviations: CVH, cardiovascular health; HPFS, Health Professional’s Follow-up Study; LE8, Life’s Essential 8; NHS, Nurses’ Health Study; NHSII, Nurses’ Health Study II. ^a^ Set 1 (base model): Age, sex, race/ethnicity, BMI, smoking, hypertension, hypercholesterolemia, and diabetes  Set 2: + physical activity  Set 3: + diet  Set 4: + blood pressure  Set 5: + sleep health  Set 6: + physical activity + diet  Set 7: + physical activity + blood pressure  Set 8: + physical activity + sleep health  Set 9: + diet + blood pressure  Set 10: + diet + sleep health  Set 11: + blood pressure + sleep health  Set 12: + physical activity + diet + blood pressure  Set 13: + physical activity + diet + sleep health  Set 14: + physical activity + blood pressure + sleep health  Set 15: + diet + blood pressure + sleep health  Set 16: + physical activity + diet + blood pressure + sleep health | | | | | |

**Supplemental Table 4.** Optimal hyperparameters of predictive models of CVH based on LE8 tuned by cross-validation in the training set using the NHANES (n=27,194).

| Outcomes and Predictors^a^ | Number of Iteration | Learning rate | Tree depth | Border count | L2 regularization |
| --- | --- | --- | --- | --- | --- |
| LE8 score |  |  |  |  |  |
| Predictor Set 1 | 5563 | 0.005 | 6 | 64 | 5 |
| Predictor Set 2 | 1766 | 0.01 | 6 | 64 | 1 |
| Predictor Set 3 | 2364 | 0.01 | 6 | 32 | 1 |
| Predictor Set 4 | 359 | 0.05 | 6 | 16 | 0.5 |
| Predictor Set 5 | 2643 | 0.01 | 6 | 64 | 5 |
| Predictor Set 6 | 3943 | 0.005 | 6 | 32 | 1 |
| Predictor Set 7 | 354 | 0.05 | 6 | 64 | 0.5 |
| Predictor Set 8 | 3901 | 0.005 | 6 | 32 | 0.5 |
| Predictor Set 9 | 2616 | 0.01 | 6 | 32 | 5 |
| Predictor Set 10 | 2669 | 0.01 | 6 | 32 | 5 |
| Predictor Set 11 | 2057 | 0.01 | 6 | 64 | 1 |
| Predictor Set 12 | 5895 | 0.005 | 6 | 64 | 5 |
| Predictor Set 13 | 4269 | 0.005 | 6 | 64 | 0.5 |
| Predictor Set 14 | 1983 | 0.01 | 6 | 32 | 0.5 |
| Predictor Set 15 | 1943 | 0.01 | 6 | 32 | 0.1 |
| Predictor Set 16 | 4617 | 0.005 | 6 | 64 | 0.1 |
| High vs. Moderate/Low CVH |  |  |  |  |  |
| Predictor Set 1 | 2573 | 0.01 | 6 | 16 | 1 |
| Predictor Set 2 | 1334 | 0.01 | 6 | 64 | 0.1 |
| Predictor Set 3 | 450 | 0.05 | 6 | 16 | 0.5 |
| Predictor Set 4 | 1423 | 0.01 | 7 | 16 | 0.5 |
| Predictor Set 5 | 1499 | 0.01 | 6 | 16 | 0.1 |
| Predictor Set 6 | 208 | 0.05 | 6 | 64 | 0.5 |
| Predictor Set 7 | 1503 | 0.01 | 6 | 64 | 0.5 |
| Predictor Set 8 | 2171 | 0.01 | 6 | 32 | 0.5 |
| Predictor Set 9 | 4011 | 0.005 | 6 | 32 | 0.5 |
| Predictor Set 10 | 1366 | 0.01 | 7 | 32 | 0.5 |
| Predictor Set 11 | 2714 | 0.005 | 6 | 16 | 0.1 |
| Predictor Set 12 | 1959 | 0.01 | 6 | 64 | 1 |
| Predictor Set 13 | 206 | 0.05 | 7 | 64 | 0.1 |
| Predictor Set 14 | 1760 | 0.01 | 6 | 32 | 1 |
| Predictor Set 15 | 2812 | 0.005 | 6 | 32 | 0.1 |
| Predictor Set 16 | 2548 | 0.005 | 6 | 16 | 0.1 |
| Low vs. Moderate/High CVH |  |  |  |  |  |
| Predictor Set 1 | 236 | 0.05 | 6 | 64 | 1 |
| Predictor Set 2 | 1457 | 0.01 | 6 | 16 | 0.5 |
| Predictor Set 3 | 2879 | 0.005 | 6 | 64 | 1 |
| Predictor Set 4 | 2444 | 0.01 | 6 | 16 | 5 |
| Predictor Set 5 | 4435 | 0.005 | 6 | 16 | 1 |
| Predictor Set 6 | 2781 | 0.005 | 6 | 16 | 1 |
| Predictor Set 7 | 2961 | 0.005 | 6 | 16 | 0.5 |
| Predictor Set 8 | 3748 | 0.005 | 6 | 32 | 1 |
| Predictor Set 9 | 1953 | 0.01 | 6 | 64 | 5 |
| Predictor Set 10 | 3511 | 0.005 | 6 | 32 | 1 |
| Predictor Set 11 | 1091 | 0.05 | 6 | 16 | 5 |
| Predictor Set 12 | 1790 | 0.01 | 6 | 16 | 0.1 |
| Predictor Set 13 | 2177 | 0.01 | 6 | 32 | 1 |
| Predictor Set 14 | 361 | 0.05 | 6 | 32 | 1 |
| Predictor Set 15 | 3496 | 0.005 | 6 | 16 | 1 |
| Predictor Set 16 | 426 | 0.05 | 6 | 32 | 0.5 |
| Abbreviations: CVH, cardiovascular health; LE8, Life’s Essential 8; NHANES: the National Health and Nutrition Examination Survey. ^a^ Set 1 (base model): Age, sex, race/ethnicity, BMI, smoking, hypertension, hypercholesterolemia, and diabetes  Set 2: + physical activity  Set 3: + diet  Set 4: + blood pressure  Set 5: + sleep health  Set 6: + physical activity + diet  Set 7: + physical activity + blood pressure  Set 8: + physical activity + sleep health  Set 9: + diet + blood pressure  Set 10: + diet + sleep health  Set 11: + blood pressure + sleep health  Set 12: + physical activity + diet + blood pressure  Set 13: + physical activity + diet + sleep health  Set 14: + physical activity + blood pressure + sleep health  Set 15: + diet + blood pressure + sleep health  Set 16: + physical activity + diet + blood pressure + sleep health | | | | | |

**Supplemental Table 5.** Performance of models to estimate overall CVH based on LE8 using NHS, NHSII, and HPFS (n=5,588).

| **Predictors^a^** | **LE8 Score** | | |  | **High vs. Moderate/Low CVH** | | |  | **Low vs. Moderate/High CVH** | | |
| --- | --- | --- | --- | --- | --- | --- | --- | --- | --- | --- | --- |
|  | RMSE | | |  | AUC | | |  | AUC | | |
|  | CV | IV | EV |  | CV | IV | EV |  | CV | IV | EV |
| Predictor Set 1 | 8.20 | 8.06 | 16.72 |  | 0.90 | 0.91 | 0.56 |  | 0.92 | 0.92 | 0.60 |
| Predictor Set 2 | 6.99 | 6.87 | 16.88 |  | 0.93 | 0.92 | 0.76 |  | 0.95 | 0.96 | 0.75 |
| Predictor Set 3 | 6.76 | 6.69 | 18.87 |  | 0.94 | 0.95 | 0.69 |  | 0.94 | 0.95 | 0.62 |
| Predictor Set 4 | 7.48 | 7.40 | 14.53 |  | 0.93 | 0.94 | 0.74 |  | 0.93 | 0.93 | 0.64 |
| Predictor Set 5 | 7.76 | 7.60 | 16.36 |  | 0.91 | 0.92 | 0.69 |  | 0.93 | 0.93 | 0.66 |
| Predictor Set 6 | 5.66 | 5.50 | 20.11 |  | 0.95 | 0.96 | 0.78 |  | 0.97 | 0.97 | 0.73 |
| Predictor Set 7 | 6.20 | 6.18 | 15.43 |  | 0.95 | 0.94 | 0.87 |  | 0.96 | 0.96 | 0.78 |
| Predictor Set 8 | 6.51 | 6.38 | 17.98 |  | 0.93 | 0.93 | 0.80 |  | 0.96 | 0.96 | 0.77 |
| Predictor Set 9 | 5.97 | 5.98 | 16.67 |  | 0.96 | 0.97 | 0.79 |  | 0.95 | 0.95 | 0.68 |
| Predictor Set 10 | 6.20 | 6.14 | 18.86 |  | 0.95 | 0.95 | 0.71 |  | 0.95 | 0.95 | 0.63 |
| Predictor Set 11 | 7.06 | 6.88 | 14.25 |  | 0.94 | 0.95 | 0.78 |  | 0.94 | 0.94 | 0.72 |
| Predictor Set 12 | 4.76 | 4.73 | 18.35 |  | 0.97 | 0.97 | 0.85 |  | 0.97 | 0.98 | 0.76 |
| Predictor Set 13 | 5.03 | 4.92 | 20.18 |  | 0.96 | 0.96 | 0.78 |  | 0.98 | 0.98 | 0.76 |
| Predictor Set 14 | 5.72 | 5.62 | 15.28 |  | 0.95 | 0.95 | 0.88 |  | 0.97 | 0.97 | 0.81 |
| Predictor Set 15 | 5.41 | 5.28 | 16.94 |  | 0.97 | 0.97 | 0.81 |  | 0.96 | 0.97 | 0.70 |
| Predictor Set 16 | 4.07 | 3.94 | 18.51 |  | 0.98 | 0.98 | 0.89 |  | 0.98 | 0.98 | 0.78 |
| Abbreviations: AUC, area under the receiver operator characteristic curve; BMI, body mass index; CV, cross-validation; CVH, cardiovascular health; EV, external validation; HPFS, Health Professional’s Follow-up Study; IV, internal validation; LE8, Life’s Essential 8; NHS, Nurses’ Health Study; NHSII, Nurses’ Health Study II; RMSE, root mean square error. ^a^ Set 1 (base model): Age, sex, race/ethnicity, BMI, smoking, hypertension, hypercholesterolemia, and diabetes  Set 2: + physical activity  Set 3: + diet  Set 4: + blood pressure  Set 5: + sleep health  Set 6: + physical activity + diet  Set 7: + physical activity + blood pressure  Set 8: + physical activity + sleep health  Set 9: + diet + blood pressure  Set 10: + diet + sleep health  Set 11: + blood pressure + sleep health  Set 12: + physical activity + diet + blood pressure  Set 13: + physical activity + diet + sleep health  Set 14: + physical activity + blood pressure + sleep health  Set 15: + diet + blood pressure + sleep health  Set 16: + physical activity + diet + blood pressure + sleep health | | | | | | | | | | | |

**Supplemental Table 6.** Performance of models to estimate overall CVH based on LE8 using the 2005-2016 NHANES (n=27,194).

| **Predictors^a^** | **LE8 Score** | | |  | **High vs. Moderate/Low CVH** | | |  | **Low vs. Moderate/High CVH** | | |
| --- | --- | --- | --- | --- | --- | --- | --- | --- | --- | --- | --- |
|  | RMSE | | |  | AUC | | |  | AUC | | |
|  | CV | IV | EV |  | CV | IV | EV |  | CV | IV | EV |
| Predictor Set 1 | 9.19 | 9.21 | 18.33 |  | 0.91 | 0.91 | 0.70 |  | 0.88 | 0.89 | 0.51 |
| Predictor Set 2 | 7.01 | 7.13 | 14.18 |  | 0.95 | 0.95 | 0.75 |  | 0.93 | 0.93 | 0.65 |
| Predictor Set 3 | 8.59 | 8.62 | 14.90 |  | 0.93 | 0.92 | 0.75 |  | 0.90 | 0.91 | 0.55 |
| Predictor Set 4 | 8.53 | 8.52 | 18.62 |  | 0.92 | 0.93 | 0.79 |  | 0.90 | 0.91 | 0.60 |
| Predictor Set 5 | 8.55 | 8.60 | 17.97 |  | 0.92 | 0.92 | 0.72 |  | 0.90 | 0.90 | 0.59 |
| Predictor Set 6 | 6.22 | 6.32 | 11.62 |  | 0.97 | 0.97 | 0.80 |  | 0.94 | 0.95 | 0.70 |
| Predictor Set 7 | 6.13 | 6.23 | 13.34 |  | 0.97 | 0.97 | 0.84 |  | 0.95 | 0.95 | 0.73 |
| Predictor Set 8 | 6.33 | 6.45 | 13.17 |  | 0.96 | 0.96 | 0.77 |  | 0.95 | 0.94 | 0.69 |
| Predictor Set 9 | 7.86 | 7.87 | 14.84 |  | 0.94 | 0.95 | 0.81 |  | 0.92 | 0.92 | 0.64 |
| Predictor Set 10 | 7.87 | 7.93 | 14.19 |  | 0.94 | 0.93 | 0.75 |  | 0.92 | 0.92 | 0.62 |
| Predictor Set 11 | 7.82 | 7.84 | 17.38 |  | 0.93 | 0.94 | 0.81 |  | 0.92 | 0.92 | 0.66 |
| Predictor Set 12 | 5.19 | 5.29 | 10.82 |  | 0.98 | 0.98 | 0.87 |  | 0.96 | 0.96 | 0.76 |
| Predictor Set 13 | 5.40 | 5.49 | 11.59 |  | 0.98 | 0.97 | 0.82 |  | 0.96 | 0.96 | 0.71 |
| Predictor Set 14 | 5.33 | 5.41 | 12.67 |  | 0.97 | 0.97 | 0.85 |  | 0.96 | 0.96 | 0.74 |
| Predictor Set 15 | 7.05 | 7.09 | 13.61 |  | 0.95 | 0.95 | 0.83 |  | 0.94 | 0.94 | 0.70 |
| Predictor Set 16 | 4.16 | 4.24 | 10.39 |  | 0.99 | 0.99 | 0.89 |  | 0.97 | 0.97 | 0.77 |
| Abbreviations: AUC, area under the receiver operator characteristic curve; BMI, body mass index; CV, cross-validation; CVH, cardiovascular health; EV, external validation; LE8, Life’s Essential 8; NHANES: the National Health and Nutrition Examination Survey; RMSE, root mean square error. ^a^ Set 1 (base model): Age, sex, race/ethnicity, BMI, smoking, hypertension, hypercholesterolemia, and diabetes  Set 2: + physical activity  Set 3: + diet  Set 4: + blood pressure  Set 5: + sleep health  Set 6: + physical activity + diet  Set 7: + physical activity + blood pressure  Set 8: + physical activity + sleep health  Set 9: + diet + blood pressure  Set 10: + diet + sleep health  Set 11: + blood pressure + sleep health  Set 12: + physical activity + diet + blood pressure  Set 13: + physical activity + diet + sleep health  Set 14: + physical activity + blood pressure + sleep health  Set 15: + diet + blood pressure + sleep health  Set 16: + physical activity + diet + blood pressure + sleep health | | | | | | | | | | | |

**Supplemental Table 7.** Internal validation of models to estimate overall CVH based on LE8 in the testing sets of NHS, NHSII, and HPFS (n=5,588).

| **Predictors** | **LE8 Score** | | |  | **High vs. Moderate/Low CVH** | | |  | **Low vs. Moderate/High CVH** | | |
| --- | --- | --- | --- | --- | --- | --- | --- | --- | --- | --- | --- |
|  | RMSE | | |  | AUC | | |  | AUC | | |
|  | NHS | NHSII | HPFS |  | NHS | NHSII | HPFS |  | NHS | NHSII | HPFS |
| Predictor Set 1 | 8.21 | 8.28 | 6.94 |  | 0.88 | 0.92 | 0.93 |  | 0.92 | 0.97 | 0.91 |
| Predictor Set 2 | 6.98 | 7.01 | 6.15 |  | 0.90 | 0.90 | 0.86 |  | 0.96 | 0.97 | 0.95 |
| Predictor Set 3 | 6.85 | 6.90 | 5.55 |  | 0.93 | 0.93 | 0.96 |  | 0.94 | 0.98 | 0.93 |
| Predictor Set 4 | 7.55 | 7.50 | 6.48 |  | 0.92 | 0.93 | 0.98 |  | 0.93 | 0.98 | 0.91 |
| Predictor Set 5 | 7.77 | 7.85 | 6.41 |  | 0.90 | 0.93 | 0.93 |  | 0.93 | 0.97 | 0.94 |
| Predictor Set 6 | 5.65 | 5.68 | 4.45 |  | 0.95 | 0.94 | 0.94 |  | 0.97 | 0.98 | 0.97 |
| Predictor Set 7 | 6.27 | 6.48 | 5.38 |  | 0.93 | 0.93 | 0.98 |  | 0.96 | 0.97 | 0.95 |
| Predictor Set 8 | 6.50 | 6.44 | 5.56 |  | 0.91 | 0.92 | 0.93 |  | 0.96 | 0.98 | 0.96 |
| Predictor Set 9 | 6.13 | 5.97 | 5.17 |  | 0.96 | 0.97 | 0.99 |  | 0.95 | 0.98 | 0.94 |
| Predictor Set 10 | 6.33 | 6.24 | 4.94 |  | 0.94 | 0.95 | 0.94 |  | 0.95 | 0.97 | 0.97 |
| Predictor Set 11 | 7.00 | 7.13 | 5.93 |  | 0.93 | 0.94 | 0.99 |  | 0.94 | 0.98 | 0.95 |
| Predictor Set 12 | 4.85 | 4.85 | 3.88 |  | 0.97 | 0.97 | 0.99 |  | 0.97 | 0.98 | 0.97 |
| Predictor Set 13 | 5.12 | 4.71 | 3.91 |  | 0.95 | 0.96 | 0.96 |  | 0.97 | 0.98 | 0.98 |
| Predictor Set 14 | 5.69 | 5.94 | 4.92 |  | 0.94 | 0.94 | 0.99 |  | 0.97 | 0.98 | 0.97 |
| Predictor Set 15 | 5.44 | 5.13 | 4.47 |  | 0.96 | 0.98 | 0.99 |  | 0.96 | 0.98 | 0.97 |
| Predictor Set 16 | 4.10 | 3.65 | 3.23 |  | 0.98 | 0.98 | 0.99 |  | 0.98 | 0.98 | 0.99 |
| Abbreviations: AUC, area under the receiver operator characteristic curve; BMI, body mass index; CV, cross-validation; CVH, cardiovascular health; EV, external validation; HPFS, Health Professional’s Follow-up Study; IV, internal validation; LE8, Life’s Essential 8; NHS, Nurses’ Health Study; NHSII, Nurses’ Health Study II; RMSE, root mean square error. ^a^ Set 1 (base model): Age, gender, race/ethnicity, BMI, smoking, hypertension, hypercholesterolemia, and diabetes  Set 2: + physical activity  Set 3: + diet  Set 4: + blood pressure  Set 5: + sleep health  Set 6: + physical activity + diet  Set 7: + physical activity + blood pressure  Set 8: + physical activity + sleep health  Set 9: + diet + blood pressure  Set 10: + diet + sleep health  Set 11: + blood pressure + sleep health  Set 12: + physical activity + diet + blood pressure  Set 13: + physical activity + diet + sleep health  Set 14: + physical activity + blood pressure + sleep health  Set 15: + diet + blood pressure + sleep health  Set 16: + physical activity + diet + blood pressure + sleep health | | | | | | | | | | | |

**Supplemental Table 8.** Characteristics of participants in NHS, NHSII, and HPFS, and the 1999-2016 NHANES included in developing prediction models of Life’s Simple 7 score.

| **Characteristics** | **NHS, NHSII, and HPFS Cohorts** | | | | **NHANES** |
| --- | --- | --- | --- | --- | --- |
|  | NHS | NHSII | HPFS | Total |  |
|  | (n=5,369) | (n=2,032) | (n=1,099) | (n=8,500) | (n=39,933) |
|  | Mean ± SD / n (%) | | | | |
| **Age (years)** | 59.06 ± 6.60 | 45.78 ± 4.17 | 63.59 ± 8.64 | 56.47 ± 8.91 | 48.66 ± 18.26 |
| **Sex** |  |  |  |  |  |
| Male | 0 (0.0) | 0 (0.0) | 1,099 (100.0) | 1,099 (12.9) | 19,345 (48.4) |
| Female | 5,369 (100.0) | 2,032 (100.0) | 0 (0.0) | 7,401 (87.1) | 20,588 (51.6) |
| **Race/ethnicity** |  |  |  |  |  |
| Non-Hispanic White | 5,062 (94.3) | 1,929 (94.9) | 509 (46.3) | 7,500 (88.2) | 18,508 (46.3) |
| Non-Hispanic Black | 24 (0.4) | 29 (1.4) | 0 (0.0) | 53 (0.6) | 7,795 (19.5) |
| Hispanic | 37 (0.7) | 33 (1.6) | 5 (0.5) | 75 (0.9) | 10,623 (26.6) |
| Others | 246 (4.6) | 41 (2.0) | 585 (53.2) | 872 (10.3) | 3,007 (7.5) |
| **BMI (continuous)** | 26.32 ± 5.18 | 28.33 ± 7.07 | 25.96 ± 3.38 | 26.76 ± 5.58 | 28.80 ± 6.62 |
| **Hypertension** |  |  |  |  |  |
| No | 3,988 (74.3) | 1,689 (83.1) | 810 (73.7) | 6,487 (76.3) | 26,482 (66.3) |
| Yes | 1,381 (25.7) | 343 (16.9) | 289 (26.3) | 2,013 (23.7) | 13,299 (33.3) |
| Missing | 0 (0.0) | 0 (0.0) | 0 (0.0) | 0 (0.0) | 152 (0.4) |
| **Diabetes** |  |  |  |  |  |
| No | 4,749 (88.5) | 1,996 (98.2) | 1,031 (93.8) | 7,776 (91.5) | 34,789 (87.1) |
| Yes | 620 (11.5) | 36 (1.8) | 68 (6.2) | 724 (8.5) | 5,121 (12.8) |
| Missing | 0 (0.0) | 0 (0.0) | 0 (0.0) | 0 (0.0) | 23 (0.1) |
| **Hypercholesterolemia** |  |  |  |  |  |
| No | 3,356 (62.5) | 1,625 (80.0) | 785 (71.4) | 5,766 (67.8) | 19,426 (48.6) |
| Yes | 2,013 (37.5) | 407 (20.0) | 314 (28.6) | 2,734 (32.2) | 12,227 (30.6) |
| Missing | 0 (0.0) | 0 (0.0) | 0 (0.0) | 0 (0.0) | 8,280 (20.7) |
|  |  |  |  |  |  |
| **Overall CVH** |  |  |  |  |  |
| ***LS7 score (0-14)*** | 8.78 ± 2.24 | 9.43 ± 2.46 | 9.61 ± 1.95 | 9.04 ± 2.28 | 8.33 ± 2.38 |
| ***Number of ideal LS7 metrics*** |  |  |  |  |  |
| 0 | 68 (1.3) | 17 (0.8) | 6 (0.5) | 91 (1.1) | 744 (1.9) |
| 1 | 546 (10.2) | 195 (9.6) | 70 (6.4) | 811 (9.5) | 4,596 (11.5) |
| 2 | 1,143 (21.3) | 397 (19.5) | 161 (14.6) | 1,701 (20.0) | 8,651 (21.7) |
| 3 | 1,440 (26.8) | 412 (20.3) | 305 (27.8) | 2,157 (25.4) | 10,298 (25.8) |
| 4 | 1,232 (22.9) | 381 (18.8) | 301 (27.4) | 1,914 (22.5) | 8,505 (21.3) |
| 5 | 684 (12.7) | 352 (17.3) | 187 (17.0) | 1,223 (14.4) | 4,932 (12.4) |
| 6 | 212 (3.9) | 218 (10.7) | 60 (5.5) | 490 (5.8) | 1,871 (4.7) |
| 7 | 44 (0.8) | 60 (3.0) | 9 (0.8) | 113 (1.3) | 336 (0.8) |
|  |  |  |  |  |  |
| **Individual LS7 metrics** |  |  |  |  |  |
| ***Blood pressure*** |  |  |  |  |  |
| Poor | 1,245 (23.2) | 229 (11.3) | 216 (19.7) | 1,690 (19.9) | 6,909 (17.3) |
| Intermediate | 3,398 (63.3) | 1,254 (61.7) | 793 (72.2) | 5,445 (64.1) | 22,084 (55.3) |
| Ideal | 726 (13.5) | 549 (27.0) | 90 (8.2) | 1,365 (16.1) | 10,940 (27.4) |
| ***HbA1c*** |  |  |  |  |  |
| Poor | 658 (12.3) | 97 (4.8) | 91 (8.3) | 846 (10.0) | 4,096 (10.3) |
| Intermediate | 1,314 (24.5) | 437 (21.5) | 387 (35.2) | 2,138 (25.2) | 9,299 (23.3) |
| Ideal | 3,397 (63.3) | 1,498 (73.7) | 621 (56.5) | 5,516 (64.9) | 26,538 (66.5) |
| ***Total cholesterol*** |  |  |  |  |  |
| Poor | 1,893 (35.3) | 326 (16.0) | 149 (13.6) | 2,368 (27.9) | 5,841 (14.6) |
| Intermediate | 2,273 (42.3) | 822 (40.5) | 470 (42.8) | 3,565 (41.9) | 15,931 (39.9) |
| Ideal | 1,203 (22.4) | 884 (43.5) | 480 (43.7) | 2,567 (30.2) | 18,161 (45.5) |
| ***BMI*** |  |  |  |  |  |
| Poor | 1,114 (20.7) | 679 (33.4) | 123 (11.2) | 1,916 (22.5) | 14,261 (35.7) |
| Intermediate | 1,699 (31.6) | 532 (26.2) | 521 (47.4) | 2,752 (32.4) | 13,628 (34.1) |
| Ideal | 2,556 (47.6) | 821 (40.4) | 455 (41.4) | 3,832 (45.1) | 12,044 (30.2) |
| ***Cigarette smoking*** |  |  |  |  |  |
| Poor | 885 (16.5) | 195 (9.6) | 65 (5.9) | 1,145 (13.5) | 8,393 (21.0) |
| Intermediate | 116 (2.2) | 43 (2.1) | 65 (5.9) | 224 (2.6) | 1,142 (2.9) |
| Ideal | 4,368 (81.4) | 1,794 (88.3) | 969 (88.2) | 7,131 (83.9) | 30,398 (76.1) |
| ***Physical activity*** |  |  |  |  |  |
| Poor | 0 (0.0) | 0 (0.0) | 15 (1.4) | 15 (0.2) | 19,128 (47.9) |
| Intermediate | 2,343 (43.6) | 953 (46.9) | 247 (22.5) | 3,543 (41.7) | 7,147 (17.9) |
| Ideal | 3,026 (56.4) | 1,079 (53.1) | 837 (76.2) | 4,942 (58.1) | 13,658 (34.2) |
| ***Diet^a^*** |  |  |  |  |  |
| *Based on NHS, NHSII, and HPFS* |  |  |  |  |  |
| Poor | 1,723 (32.1) | 773 (38.0) | 339 (30.8) | 2,835 (33.4) | 35,955 (90.0) |
| Intermediate | 1,842 (34.3) | 647 (31.8) | 343 (31.2) | 2,832 (33.3) | 2,556 (6.4) |
| Ideal | 1,804 (33.6) | 612 (30.1) | 417 (37.9) | 2,833 (33.3) | 1,422 (3.6) |
| *Based on NHANES* |  |  |  |  |  |
| Poor | 192 (3.6) | 118 (5.8) | 49 (4.5) | 359 (4.2) | 13,311 (33.3) |
| Intermediate | 370 (6.9) | 170 (8.4) | 86 (7.8) | 626 (7.4) | 13,311 (33.3) |
| Ideal | 4,807 (89.5) | 1,744 (85.8) | 964 (87.7) | 7,515 (88.4) | 13,311 (33.3) |
| Abbreviations: BMI, body mass index; CVH, cardiovascular health; HbA1c, glycohemoglobin; LS7, Life’s Simple 7.  ^a^ Cut points for AHEI-2010 tertiles are 48.0 and 57.8 in the NHS, NHSII, and HPFS, and 34.3 and 39.6 in the NHANES. | | | | | |

**Supplemental Table 9.** Optimal hyperparameters of predictive models of CVH based on LS7 tuned by cross-validation in the training set using NHS, NHSII, and HPFS (n=8,500).

| Outcomes and Predictors^a^ | Number of Iteration | Learning rate | Tree depth | Border count | L2 regularization |
| --- | --- | --- | --- | --- | --- |
| ≥1 Ideal CVH metrics |  |  |  |  |  |
| Predictor Set 1 | 569 | 0.05 | 6 | 32 | 5 |
| Predictor Set 2 | 683 | 0.05 | 6 | 64 | 0.1 |
| Predictor Set 3 | 743 | 0.05 | 6 | 32 | 1 |
| Predictor Set 4 | 623 | 0.01 | 6 | 16 | 0.5 |
| Predictor Set 5 | 483 | 0.05 | 6 | 32 | 1 |
| Predictor Set 6 | 269 | 0.01 | 7 | 16 | 0.1 |
| Predictor Set 7 | 82 | 0.05 | 7 | 32 | 0.1 |
| Predictor Set 8 | 303 | 0.05 | 6 | 16 | 0.5 |
| ≥2 Ideal CVH metrics |  |  |  |  |  |
| Predictor Set 1 | 88 | 0.05 | 7 | 64 | 1 |
| Predictor Set 2 | 220 | 0.05 | 6 | 64 | 0.5 |
| Predictor Set 3 | 477 | 0.05 | 6 | 64 | 5 |
| Predictor Set 4 | 107 | 0.05 | 7 | 32 | 0.5 |
| Predictor Set 5 | 285 | 0.05 | 6 | 64 | 0.1 |
| Predictor Set 6 | 315 | 0.05 | 6 | 16 | 1 |
| Predictor Set 7 | 422 | 0.05 | 6 | 64 | 1 |
| Predictor Set 8 | 326 | 0.05 | 6 | 16 | 0.5 |
| ≥3 Ideal CVH metrics |  |  |  |  |  |
| Predictor Set 1 | 1017 | 0.01 | 6 | 64 | 0.5 |
| Predictor Set 2 | 279 | 0.05 | 6 | 64 | 5 |
| Predictor Set 3 | 1273 | 0.01 | 6 | 16 | 0.1 |
| Predictor Set 4 | 1552 | 0.01 | 6 | 32 | 1 |
| Predictor Set 5 | 2942 | 0.005 | 6 | 32 | 1 |
| Predictor Set 6 | 1901 | 0.005 | 6 | 32 | 5 |
| Predictor Set 7 | 1310 | 0.01 | 6 | 64 | 0.1 |
| Predictor Set 8 | 1091 | 0.01 | 6 | 16 | 0.5 |
| ≥4 Ideal CVH metrics |  |  |  |  |  |
| Predictor Set 1 | 128 | 0.05 | 6 | 32 | 1 |
| Predictor Set 2 | 136 | 0.05 | 6 | 32 | 1 |
| Predictor Set 3 | 297 | 0.05 | 6 | 64 | 1 |
| Predictor Set 4 | 113 | 0.05 | 6 | 64 | 1 |
| Predictor Set 5 | 293 | 0.05 | 6 | 16 | 0.5 |
| Predictor Set 6 | 108 | 0.05 | 6 | 32 | 1 |
| Predictor Set 7 | 2225 | 0.01 | 6 | 64 | 5 |
| Predictor Set 8 | 1965 | 0.01 | 6 | 16 | 1 |
| ≥5 Ideal CVH metrics |  |  |  |  |  |
| Predictor Set 1 | 259 | 0.05 | 6 | 16 | 0.5 |
| Predictor Set 2 | 1064 | 0.01 | 6 | 16 | 0.1 |
| Predictor Set 3 | 1394 | 0.01 | 7 | 32 | 0.1 |
| Predictor Set 4 | 168 | 0.05 | 6 | 64 | 1 |
| Predictor Set 5 | 2088 | 0.01 | 6 | 16 | 0.1 |
| Predictor Set 6 | 952 | 0.01 | 6 | 16 | 0.5 |
| Predictor Set 7 | 2461 | 0.005 | 6 | 32 | 0.1 |
| Predictor Set 8 | 2421 | 0.005 | 6 | 64 | 1 |
| ≥6 Ideal CVH metrics |  |  |  |  |  |
| Predictor Set 1 | 229 | 0.05 | 6 | 32 | 0.5 |
| Predictor Set 2 | 134 | 0.05 | 7 | 32 | 0.1 |
| Predictor Set 3 | 2086 | 0.05 | 6 | 16 | 5 |
| Predictor Set 4 | 378 | 0.05 | 6 | 16 | 0.5 |
| Predictor Set 5 | 184 | 0.05 | 7 | 64 | 0.1 |
| Predictor Set 6 | 162 | 0.05 | 7 | 64 | 0.5 |
| Predictor Set 7 | 108 | 0.05 | 7 | 16 | 0.5 |
| Predictor Set 8 | 1511 | 0.01 | 6 | 64 | 0.5 |
| 7 Ideal CVH metrics |  |  |  |  |  |
| Predictor Set 1 | 183 | 0.05 | 7 | 32 | 1 |
| Predictor Set 2 | 192 | 0.05 | 7 | 32 | 0.1 |
| Predictor Set 3 | 179 | 0.05 | 6 | 32 | 0.5 |
| Predictor Set 4 | 87 | 0.05 | 6 | 64 | 1 |
| Predictor Set 5 | 167 | 0.05 | 7 | 32 | 0.1 |
| Predictor Set 6 | 110 | 0.05 | 6 | 64 | 1 |
| Predictor Set 7 | 252 | 0.05 | 7 | 64 | 0.5 |
| Predictor Set 8 | 66 | 0.05 | 8 | 32 | 0.1 |
| LS7 score |  |  |  |  |  |
| Predictor Set 1 | 1702 | 0.01 | 6 | 64 | 5 |
| Predictor Set 2 | 241 | 0.05 | 8 | 16 | 5 |
| Predictor Set 3 | 4406 | 0.005 | 6 | 32 | 5 |
| Predictor Set 4 | 213 | 0.05 | 7 | 16 | 5 |
| Predictor Set 5 | 2033 | 0.01 | 6 | 64 | 5 |
| Predictor Set 6 | 1177 | 0.01 | 6 | 32 | 1 |
| Predictor Set 7 | 3436 | 0.005 | 6 | 64 | 5 |
| Predictor Set 8 | 2409 | 0.01 | 6 | 64 | 1 |
| Abbreviations: AHEI-2010: alternative healthy eating index 2010; CVH, cardiovascular health; HPFS, Health Professional’s Follow-up Study; LS7, Life’s Simple 7; NHS, Nurses’ Health Study; NHSII, Nurses’ Health Study II. ^a^ Set 1 (base model): Age, gender, race/ethnicity, BMI, smoking, hypertension, hypercholesterolemia, and diabetes  Set 2: base model + physical activity  Set 3: base model + diet (AHEI-2010)  Set 4: base model + blood pressure  Set 5: base model + physical activity + diet (AHEI-2010)  Set 6: base model + physical activity + blood pressure  Set 7: base model + diet (AHEI-2010) + blood pressure  Set 8: base model + physical activity + diet (AHEI-2010) + blood pressure | | | | | |

**Supplemental Table 10.** Optimal hyperparameters of predictive models of CVH based on LS7 tuned by cross-validation in the training set using the NHANES (n=39,933).

| Outcomes and Predictors^a^ | Number of Iteration | Learning rate | Tree depth | Border count | L2 regularization |
| --- | --- | --- | --- | --- | --- |
| ≥1 Ideal CVH metrics |  |  |  |  |  |
| Predictor Set 1 | 93 | 0.05 | 7 | 32 | 0.1 |
| Predictor Set 2 | 100 | 0.05 | 6 | 64 | 0.1 |
| Predictor Set 3 | 713 | 0.01 | 7 | 64 | 1 |
| Predictor Set 4 | 116 | 0.05 | 8 | 64 | 0.1 |
| Predictor Set 5 | 917 | 0.005 | 7 | 64 | 0.5 |
| Predictor Set 6 | 911 | 0.005 | 8 | 64 | 0.1 |
| Predictor Set 7 | 1311 | 0.01 | 8 | 64 | 1 |
| Predictor Set 8 | 452 | 0.01 | 8 | 64 | 0.5 |
| ≥2 Ideal CVH metrics |  |  |  |  |  |
| Predictor Set 1 | 3693 | 0.005 | 6 | 32 | 0.1 |
| Predictor Set 2 | 3819 | 0.005 | 6 | 32 | 0.1 |
| Predictor Set 3 | 342 | 0.05 | 6 | 32 | 0.5 |
| Predictor Set 4 | 2113 | 0.01 | 6 | 32 | 0.1 |
| Predictor Set 5 | 391 | 0.05 | 6 | 64 | 0.5 |
| Predictor Set 6 | 1911 | 0.01 | 6 | 32 | 0.5 |
| Predictor Set 7 | 1669 | 0.01 | 7 | 32 | 0.1 |
| Predictor Set 8 | 4081 | 0.005 | 7 | 32 | 1 |
| ≥3 Ideal CVH metrics |  |  |  |  |  |
| Predictor Set 1 | 1692 | 0.01 | 6 | 64 | 0.5 |
| Predictor Set 2 | 2323 | 0.01 | 6 | 32 | 1 |
| Predictor Set 3 | 1063 | 0.05 | 6 | 32 | 5 |
| Predictor Set 4 | 3895 | 0.005 | 6 | 64 | 1 |
| Predictor Set 5 | 2630 | 0.01 | 6 | 64 | 0.5 |
| Predictor Set 6 | 3526 | 0.005 | 7 | 32 | 1 |
| Predictor Set 7 | 2403 | 0.01 | 6 | 64 | 0.5 |
| Predictor Set 8 | 311 | 0.05 | 7 | 64 | 0.5 |
| ≥4 Ideal CVH metrics |  |  |  |  |  |
| Predictor Set 1 | 5189 | 0.005 | 6 | 32 | 1 |
| Predictor Set 2 | 4550 | 0.005 | 6 | 64 | 1 |
| Predictor Set 3 | 2305 | 0.01 | 6 | 64 | 1 |
| Predictor Set 4 | 3138 | 0.01 | 6 | 32 | 1 |
| Predictor Set 5 | 3628 | 0.005 | 6 | 64 | 0.1 |
| Predictor Set 6 | 383 | 0.05 | 6 | 32 | 1 |
| Predictor Set 7 | 4408 | 0.005 | 6 | 64 | 1 |
| Predictor Set 8 | 2585 | 0.01 | 7 | 32 | 1 |
| ≥5 Ideal CVH metrics |  |  |  |  |  |
| Predictor Set 1 | 651 | 0.05 | 7 | 64 | 5 |
| Predictor Set 2 | 339 | 0.05 | 8 | 32 | 5 |
| Predictor Set 3 | 3245 | 0.01 | 8 | 64 | 5 |
| Predictor Set 4 | 204 | 0.05 | 7 | 64 | 0.5 |
| Predictor Set 5 | 3850 | 0.005 | 6 | 64 | 0.1 |
| Predictor Set 6 | 415 | 0.05 | 8 | 64 | 5 |
| Predictor Set 7 | 371 | 0.05 | 6 | 64 | 0.5 |
| Predictor Set 8 | 887 | 0.05 | 6 | 64 | 5 |
| ≥6 Ideal CVH metrics |  |  |  |  |  |
| Predictor Set 1 | 1638 | 0.01 | 6 | 32 | 0.1 |
| Predictor Set 2 | 1179 | 0.01 | 6 | 32 | 0.1 |
| Predictor Set 3 | 1247 | 0.01 | 6 | 32 | 0.5 |
| Predictor Set 4 | 339 | 0.05 | 6 | 16 | 0.1 |
| Predictor Set 5 | 245 | 0.05 | 7 | 32 | 0.5 |
| Predictor Set 6 | 1909 | 0.01 | 7 | 16 | 0.1 |
| Predictor Set 7 | 258 | 0.05 | 6 | 16 | 0.1 |
| Predictor Set 8 | 1559 | 0.01 | 6 | 16 | 0.5 |
| 7 Ideal CVH metrics |  |  |  |  |  |
| Predictor Set 1 | 382 | 0.05 | 7 | 16 | 0.5 |
| Predictor Set 2 | 562 | 0.05 | 7 | 16 | 1 |
| Predictor Set 3 | 243 | 0.05 | 7 | 16 | 0.5 |
| Predictor Set 4 | 391 | 0.05 | 7 | 16 | 0.5 |
| Predictor Set 5 | 188 | 0.05 | 8 | 16 | 0.1 |
| Predictor Set 6 | 298 | 0.05 | 6 | 16 | 0.1 |
| Predictor Set 7 | 208 | 0.05 | 8 | 32 | 1 |
| Predictor Set 8 | 210 | 0.05 | 8 | 16 | 0.5 |
| LS7 score |  |  |  |  |  |
| Predictor Set 1 | 551 | 0.05 | 6 | 32 | 0.5 |
| Predictor Set 2 | 850 | 0.05 | 6 | 64 | 5 |
| Predictor Set 3 | 6403 | 0.005 | 6 | 64 | 5 |
| Predictor Set 4 | 544 | 0.05 | 6 | 32 | 1 |
| Predictor Set 5 | 831 | 0.05 | 6 | 32 | 5 |
| Predictor Set 6 | 3534 | 0.01 | 6 | 32 | 5 |
| Predictor Set 7 | 6494 | 0.005 | 6 | 16 | 1 |
| Predictor Set 8 | 3301 | 0.01 | 6 | 32 | 0.5 |
| Abbreviations: CVH, cardiovascular health; LS7, Life’s Simple 7; NHANES: the National Health and Nutrition Examination Survey. ^a^ Set 1 (base model): Age, sex, race/ethnicity, BMI, smoking, hypertension, hypercholesterolemia, and diabetes  Set 2: + physical activity  Set 3: + diet  Set 4: + blood pressure  Set 5: + physical activity + diet  Set 6: + physical activity + blood pressure  Set 7: + diet + blood pressure  Set 8: + physical activity + diet + blood pressure | | | | | |

**Supplemental Table 11.** Performance of models to estimate overall CVH based on LS7 using NHS, NHSII, and HPFS (n=8,500).

| **Predictors^a^** | **Ideal CVH (number of ideal metrics)** | | | | | | | | | | | | | | | | | | | | | **LS7 score**  **RMSE** | | |
| --- | --- | --- | --- | --- | --- | --- | --- | --- | --- | --- | --- | --- | --- | --- | --- | --- | --- | --- | --- | --- | --- | --- | --- | --- |
|  | **AUC** | | | | | | | | | | | | | | | | | | | | |  |  |  |
|  | **≥1** | | | **≥2** | | | **≥3** | | | **≥4** | | | **≥5** | | | **≥6** | | | **7** | | |  |  |  |
|  | CV | IV | EV | CV | IV | EV | CV | IV | EV | CV | IV | EV | CV | IV | EV | CV | IV | EV | CV | IV | EV | CV | IV | EV |
| Predictor Set 1 | 0.98 | 0.98 | 0.74 | 0.88 | 0.89 | 0.76 | 0.87 | 0.85 | 0.77 | 0.86 | 0.85 | 0.77 | 0.89 | 0.86 | 0.82 | 0.91 | 0.88 | 0.89 | 0.94 | 0.93 | 0.90 | 1.43 | 1.47 | 2.37 |
| Predictor Set 2 | 0.99 | 0.99 | 0.74 | 0.94 | 0.95 | 0.77 | 0.92 | 0.92 | 0.79 | 0.91 | 0.90 | 0.83 | 0.92 | 0.90 | 0.89 | 0.93 | 0.91 | 0.90 | 0.96 | 0.96 | 0.92 | 1.31 | 1.33 | 1.81 |
| Predictor Set 3 | 0.99 | 0.99 | 0.78 | 0.92 | 0.93 | 0.74 | 0.91 | 0.90 | 0.76 | 0.91 | 0.91 | 0.79 | 0.93 | 0.92 | 0.82 | 0.95 | 0.93 | 0.82 | 0.98 | 0.93 | 0.92 | 1.11 | 1.16 | 2.33 |
| Predictor Set 4 | 0.98 | 0.98 | 0.77 | 0.89 | 0.90 | 0.78 | 0.88 | 0.87 | 0.81 | 0.88 | 0.87 | 0.83 | 0.91 | 0.90 | 0.86 | 0.95 | 0.93 | 0.92 | 0.98 | 0.97 | 0.88 | 1.31 | 1.33 | 2.04 |
| Predictor Set 5 | 0.99 | 0.99 | 0.78 | 0.96 | 0.97 | 0.80 | 0.95 | 0.95 | 0.79 | 0.95 | 0.94 | 0.83 | 0.96 | 0.94 | 0.84 | 0.96 | 0.95 | 0.87 | 0.98 | 0.96 | 0.89 | 1.01 | 1.04 | 3.08 |
| Predictor Set 6 | 0.99 | 0.99 | 0.77 | 0.95 | 0.96 | 0.81 | 0.92 | 0.93 | 0.85 | 0.92 | 0.92 | 0.85 | 0.94 | 0.93 | 0.90 | 0.96 | 0.95 | 0.92 | 0.98 | 0.98 | 0.89 | 1.18 | 1.19 | 1.96 |
| Predictor Set 7 | 0.99 | 0.98 | 0.79 | 0.92 | 0.94 | 0.78 | 0.92 | 0.91 | 0.80 | 0.93 | 0.92 | 0.82 | 0.96 | 0.95 | 0.85 | 0.98 | 0.97 | 0.88 | 0.99 | 0.99 | 0.88 | 0.97 | 0.99 | 2.16 |
| Predictor Set 8 | 0.99 | 0.99 | 0.79 | 0.97 | 0.97 | 0.80 | 0.96 | 0.96 | 0.82 | 0.96 | 0.96 | 0.84 | 0.98 | 0.97 | 0.84 | 0.99 | 0.99 | 0.88 | 1.00 | 0.96 | 0.94 | 0.85 | 0.86 | 3.00 |
| Abbreviations: AUC, area under the receiver operator characteristic curve; BMI, body mass index; CV, cross-validation; CVH, cardiovascular health; EV, external validation; HPFS, Health Professional’s Follow-up Study; IV, internal validation; LS7, Life’s Simple 7; NHS, Nurses’ Health Study; NHSII, Nurses’ Health Study II; RMSE, root mean square error. ^a^ Set 1 (base model): Age, sex, race/ethnicity, BMI, smoking, hypertension, hypercholesterolemia, and diabetes  Set 2: + physical activity  Set 3: + diet  Set 4: + blood pressure  Set 5: + physical activity + diet  Set 6: + physical activity + blood pressure  Set 7: + diet + blood pressure  Set 8: + physical activity + diet + blood pressure | | | | | | | | | | | | | | | | | | | | | | | | |

**Supplemental Table 12.** Performance of models to estimate overall CVH based on LS7 using the 1999-2016 NHANES (n=39,933).

| **Predictors^a^** | **Ideal CVH (number of ideal metrics)** | | | | | | | | | | | | | | | | | | | | | **LS7 score**  **RMSE** | | |
| --- | --- | --- | --- | --- | --- | --- | --- | --- | --- | --- | --- | --- | --- | --- | --- | --- | --- | --- | --- | --- | --- | --- | --- | --- |
|  | **AUC** | | | | | | | | | | | | | | | | | | | | |  |  |  |
|  | **≥1** | | | **≥2** | | | **≥3** | | | **≥4** | | | **≥5** | | | **≥6** | | | **7** | | |  |  |  |
|  | CV | IV | EV | CV | IV | EV | CV | IV | EV | CV | IV | EV | CV | IV | EV | CV | IV | EV | CV | IV | EV | CV | IV | EV |
| Predictor Set 1 | 0.97 | 0.97 | 0.79 | 0.88 | 0.88 | 0.81 | 0.86 | 0.85 | 0.77 | 0.86 | 0.86 | 0.79 | 0.89 | 0.90 | 0.82 | 0.92 | 0.93 | 0.87 | 0.95 | 0.95 | 0.86 | 1.56 | 1.55 | 3.19 |
| Predictor Set 2 | 0.98 | 0.98 | 0.80 | 0.91 | 0.91 | 0.84 | 0.89 | 0.90 | 0.84 | 0.90 | 0.90 | 0.85 | 0.93 | 0.93 | 0.86 | 0.96 | 0.96 | 0.91 | 0.98 | 0.98 | 0.88 | 1.28 | 1.27 | 2.08 |
| Predictor Set 3 | 0.98 | 0.98 | 0.95 | 0.93 | 0.92 | 0.83 | 0.90 | 0.89 | 0.83 | 0.90 | 0.90 | 0.84 | 0.92 | 0.93 | 0.87 | 0.95 | 0.96 | 0.90 | 0.99 | 0.99 | 0.88 | 1.33 | 1.33 | 2.48 |
| Predictor Set 4 | 0.98 | 0.97 | 0.79 | 0.90 | 0.89 | 0.83 | 0.88 | 0.87 | 0.83 | 0.89 | 0.89 | 0.83 | 0.92 | 0.92 | 0.85 | 0.95 | 0.95 | 0.89 | 0.97 | 0.97 | 0.95 | 1.45 | 1.44 | 2.92 |
| Predictor Set 5 | 0.99 | 0.99 | 0.98 | 0.95 | 0.95 | 0.87 | 0.93 | 0.93 | 0.86 | 0.93 | 0.94 | 0.89 | 0.96 | 0.96 | 0.90 | 0.98 | 0.98 | 0.89 | 1.00 | 0.98 | 0.90 | 1.01 | 1.01 | 2.11 |
| Predictor Set 6 | 0.98 | 0.98 | 0.98 | 0.93 | 0.93 | 0.85 | 0.92 | 0.91 | 0.86 | 0.93 | 0.93 | 0.86 | 0.95 | 0.95 | 0.87 | 0.97 | 0.98 | 0.93 | 0.99 | 0.99 | 0.97 | 1.15 | 1.14 | 1.92 |
| Predictor Set 7 | 0.99 | 0.99 | 0.98 | 0.94 | 0.94 | 0.85 | 0.92 | 0.91 | 0.85 | 0.93 | 0.92 | 0.86 | 0.95 | 0.95 | 0.89 | 0.98 | 0.98 | 0.93 | 0.99 | 0.99 | 0.92 | 1.19 | 1.20 | 2.23 |
| Predictor Set 8 | 0.99 | 0.99 | 0.98 | 0.96 | 0.96 | 0.89 | 0.95 | 0.95 | 0.89 | 0.96 | 0.96 | 0.90 | 0.98 | 0.98 | 0.91 | 0.99 | 0.99 | 0.94 | 0.99 | 0.99 | 0.97 | 0.81 | 0.82 | 2.07 |
| Abbreviations: AUC, area under the receiver operator characteristic curve; BMI, body mass index; CV, cross-validation; CVH, cardiovascular health; EV, external validation; IV, internal validation; LS7, Life’s Simple 7; NHANES: the National Health and Nutrition Examination Survey; RMSE, root mean square error. ^a^ Set 1 (base model): Age, sex, race/ethnicity, BMI, smoking, hypertension, hypercholesterolemia, and diabetes  Set 2: + physical activity  Set 3: + diet  Set 4: + blood pressure  Set 5: + physical activity + diet  Set 6: + physical activity + blood pressure  Set 7: + diet + blood pressure  Set 8: + physical activity + diet + blood pressure | | | | | | | | | | | | | | | | | | | | | | | | |

**Supplemental Table 13.** Internal validation of models to estimate overall CVH based on LS7 in the testing sets of NHS, NHSII, and HPFS (n=8,500).

| **Predictors^a^** | **Ideal CVH (number of ideal metrics)** | | | | | | | | | | | | | | | | | | | | | **LS7 score**  **RMSE** | | |
| --- | --- | --- | --- | --- | --- | --- | --- | --- | --- | --- | --- | --- | --- | --- | --- | --- | --- | --- | --- | --- | --- | --- | --- | --- |
|  | **AUC** | | | | | | | | | | | | | | | | | | | | |  |  |  |
|  | **≥1** | | | **≥2** | | | **≥3** | | | **≥4** | | | **≥5** | | | **≥6** | | | **7** | | |  |  |  |
|  | NHS | NHSII | HPFS | NHS | NHSII | HPFS | NHS | NHSII | HPFS | NHS | NHSII | HPFS | NHS | NHSII | HPFS | NHS | NHSII | HPFS | NHS | NHSII | HPFS | NHS | NHSII | HPFS |
| Predictor Set 1 | 0.97 | 0.99 | 0.99 | 0.89 | 0.89 | 0.89 | 0.85 | 0.87 | 0.80 | 0.84 | 0.89 | 0.86 | 0.86 | 0.86 | 0.85 | 0.88 | 0.85 | 0.89 | 0.92 | 0.90 | 0.94 | 1.48 | 1.47 | 1.40 |
| Predictor Set 2 | 0.98 | 0.99 | 0.99 | 0.95 | 0.95 | 0.96 | 0.92 | 0.92 | 0.90 | 0.89 | 0.93 | 0.88 | 0.90 | 0.90 | 0.87 | 0.90 | 0.90 | 0.91 | 0.95 | 0.94 | 0.95 | 1.35 | 1.31 | 1.26 |
| Predictor Set 3 | 0.98 | 0.99 | 0.99 | 0.93 | 0.92 | 0.92 | 0.90 | 0.91 | 0.87 | 0.90 | 0.92 | 0.92 | 0.91 | 0.91 | 0.92 | 0.94 | 0.90 | 0.95 | 0.92 | 0.89 | 0.94 | 1.18 | 1.13 | 1.09 |
| Predictor Set 4 | 0.97 | 0.99 | 0.99 | 0.90 | 0.91 | 0.89 | 0.86 | 0.89 | 0.82 | 0.85 | 0.91 | 0.88 | 0.90 | 0.90 | 0.86 | 0.94 | 0.91 | 0.90 | 0.98 | 0.94 | 0.99 | 1.34 | 1.32 | 1.28 |
| Predictor Set 5 | 0.99 | 0.99 | 0.99 | 0.97 | 0.96 | 0.97 | 0.95 | 0.94 | 0.94 | 0.93 | 0.95 | 0.93 | 0.94 | 0.93 | 0.93 | 0.95 | 0.94 | 0.96 | 0.95 | 0.94 | 0.96 | 1.06 | 1.00 | 0.99 |
| Predictor Set 6 | 0.98 | 0.99 | 0.99 | 0.95 | 0.95 | 0.97 | 0.93 | 0.93 | 0.91 | 0.91 | 0.95 | 0.90 | 0.94 | 0.93 | 0.90 | 0.95 | 0.95 | 0.94 | 0.99 | 0.97 | 1.00 | 1.21 | 1.15 | 1.13 |
| Predictor Set 7 | 0.97 | 0.98 | 0.99 | 0.94 | 0.93 | 0.93 | 0.91 | 0.92 | 0.87 | 0.91 | 0.95 | 0.93 | 0.95 | 0.95 | 0.93 | 0.97 | 0.95 | 0.97 | 0.99 | 0.99 | 1.00 | 1.01 | 0.94 | 0.99 |
| Predictor Set 8 | 0.99 | 0.99 | 0.99 | 0.98 | 0.97 | 0.97 | 0.96 | 0.96 | 0.94 | 0.95 | 0.97 | 0.95 | 0.97 | 0.97 | 0.95 | 0.99 | 0.98 | 0.97 | 0.96 | 0.93 | 0.99 | 0.88 | 0.80 | 0.87 |
| Abbreviations: AUC, area under the receiver operator characteristic curve; BMI, body mass index; CVH, cardiovascular health; HPFS, Health Professional’s Follow-up Study; LS7, Life’s Simple 7; NHS, Nurses' Health Study; NHSII, Nurses' Health Study II; RMSE, root mean square error;  ^a^ Set 1 (base model): Age, sex, race/ethnicity, BMI, smoking, hypertension, hypercholesterolemia, and diabetes  Set 2: + physical activity  Set 3: + diet  Set 4: + blood pressure  Set 5: + physical activity + diet  Set 6: + physical activity + blood pressure  Set 7: + diet + blood pressure  Set 8: + physical activity + diet + blood pressure | | | | | | | | | | | | | | | | | | | | | | | | |


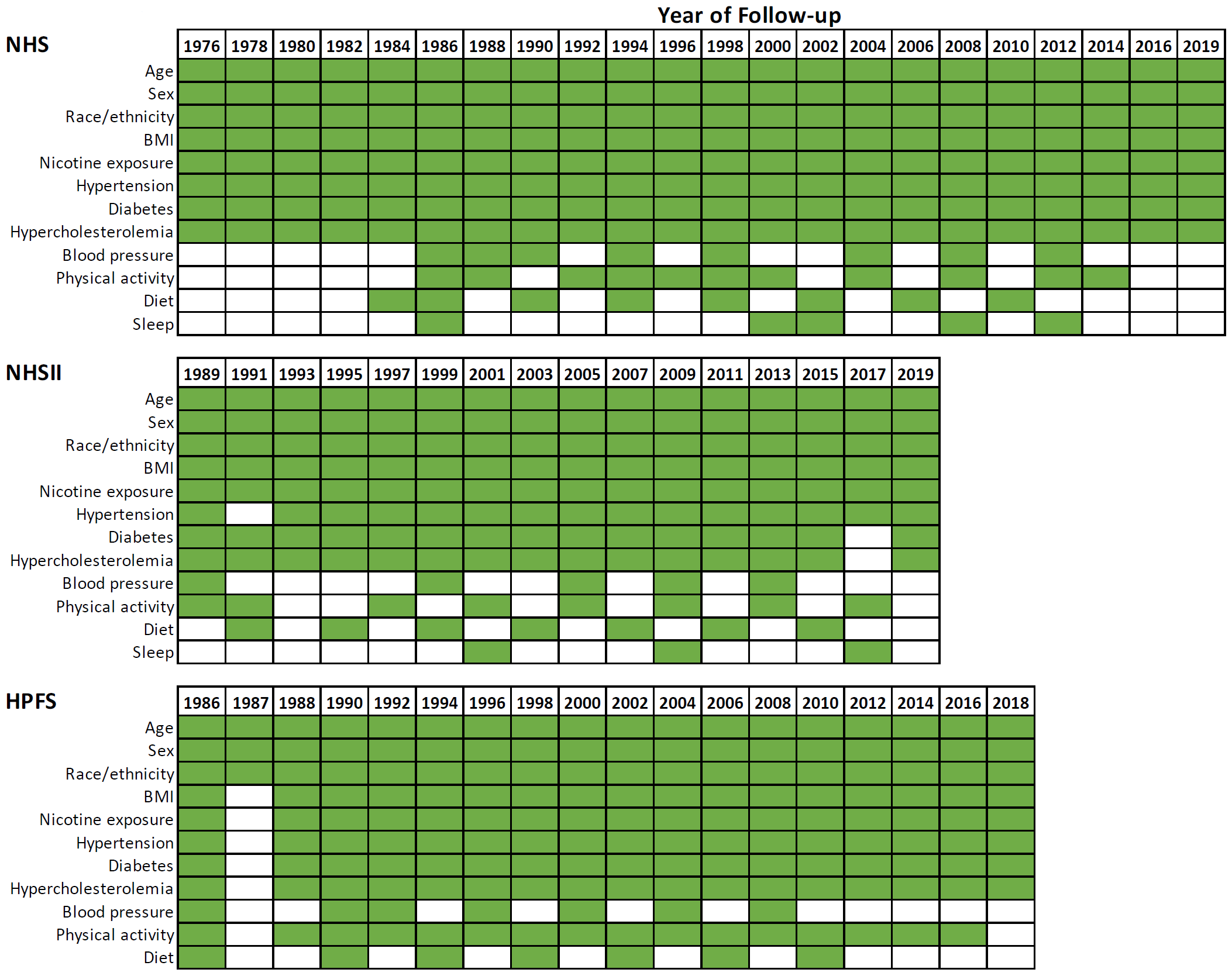


**Supplemental Figure 1.** Availabilities of CVH metrics and predictors in NHS, NHSII, and HPFS.

Abbreviations: BMI, body mass index; HPFS, Health Professional’s Follow-up Study; NHS, Nurses’ Health Study; NHSII, Nurses’ Health Study II.

**
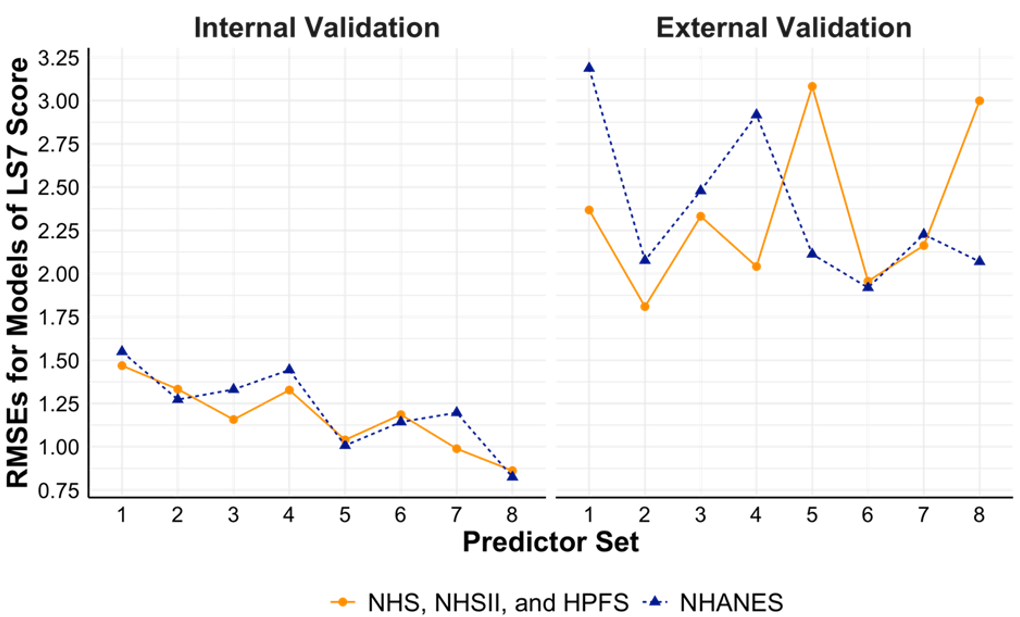
**

**Supplemental Figure 2.** Performance of models to estimate continuous overall CVH score based on LS7 using NHS, NHSII, and HPFS (n=8,500), and NHANES (n=39,933). Set 1 (base model): Age, sex, race/ethnicity, BMI, smoking, hypertension, hypercholesterolemia, and diabetes

Set 2: + physical activity

Set 3: + diet

Set 4: + blood pressure

Set 5: + physical activity + diet

Set 6: + physical activity + blood pressure

Set 7: + diet + blood pressure

Set 8: + physical activity + diet + blood pressure

Abbreviations: BMI, body mass index; CVH, cardiovascular health; HPFS, Health Professional’s Follow-up Study; LS7, Life’s Simple 7; NHANES: the National Health and Nutrition Examination Survey; NHS, Nurses’ Health Study; NHSII, Nurses’ Health Study II; RMSE, root mean square error.

**
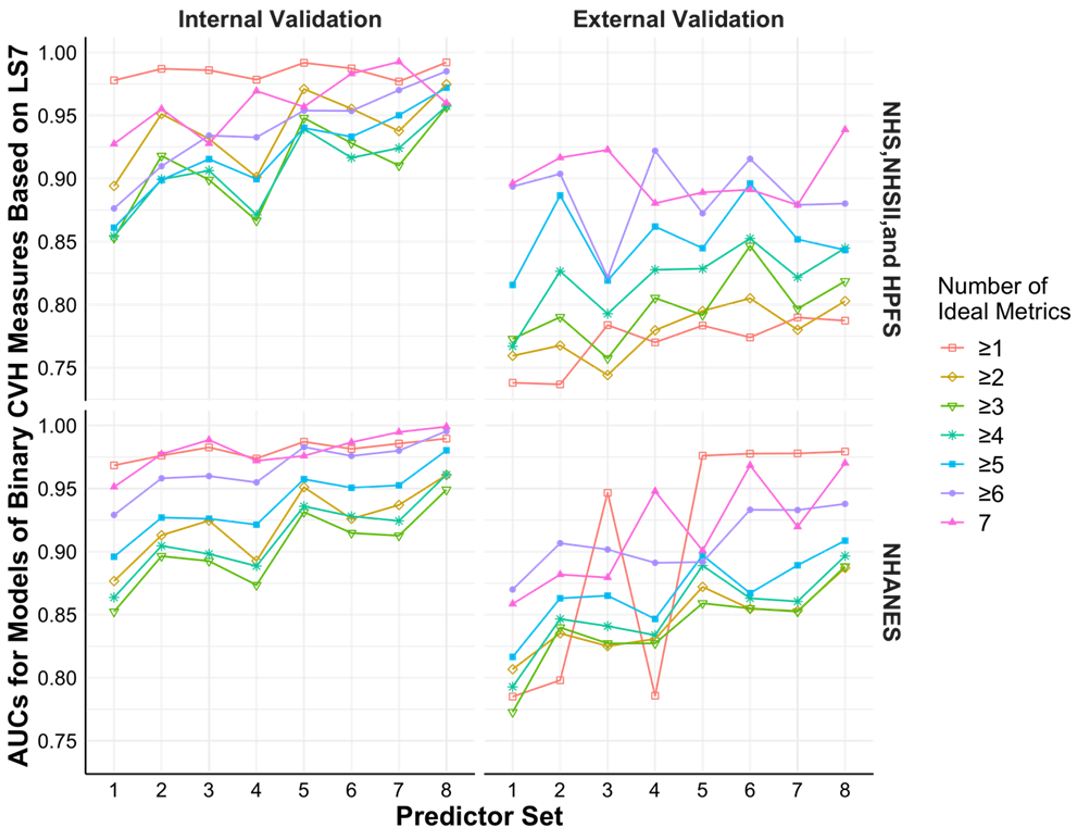
**

**Supplemental Figure 3.** Performance of models to estimate binary CVH measures based on LS7 using NHS, NHSII, and HPFS (n=8,500), and NHANES (n=39,933).

Set 1 (base model): Age, sex, race/ethnicity, BMI, smoking, hypertension, hypercholesterolemia, and diabetes;

Set 2: + physical activity

Set 3: + diet

Set 4: + blood pressure

Set 5: + physical activity + diet

Set 6: + physical activity + blood pressure

Set 7: + diet + blood pressure

Set 8: + physical activity + diet + blood pressure

Abbreviations: AUC, area under the receiver operator characteristic curve; BMI, body mass index; CVH, cardiovascular health; HPFS, Health Professional’s Follow-up Study; LS7, Life’s Simple 7; NHANES: the National Health and Nutrition Examination Survey; NHS, Nurses’ Health Study; NHSII, Nurses’ Health Study II.


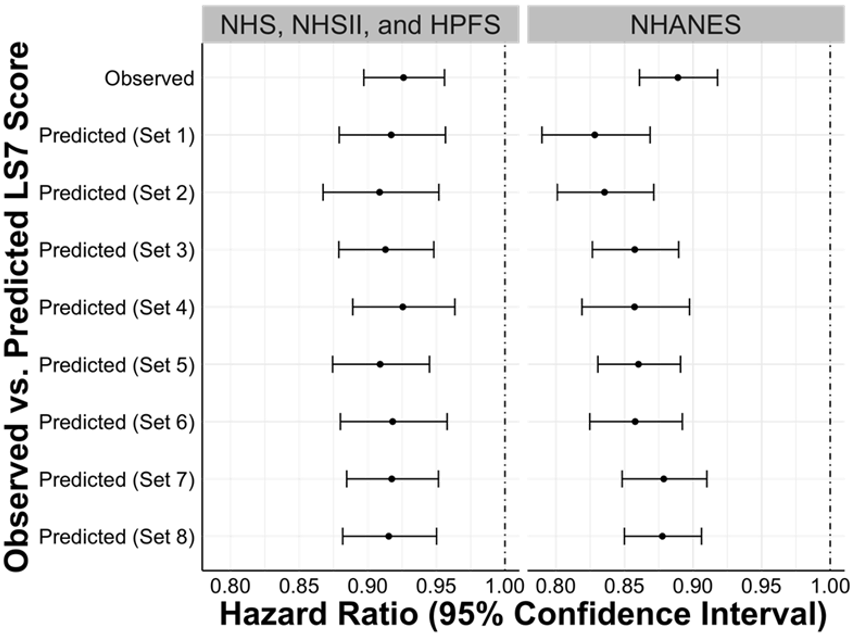


**Supplemental Figure 4.** Hazard ratios (95% confidence intervals) of all-cause mortality with original vs. predicted overall CVH score based on LS7 in testing sets of NHS, NHSII, and HPFS (n=8,500), and NHANES (n=39,933).

Set 1 (base model): Age, sex, race/ethnicity, BMI, smoking, hypertension, hypercholesterolemia, and diabetes;

Set 2: + physical activity

Set 3: + diet

Set 4: + blood pressure

Set 5: + physical activity + diet

Set 6: + physical activity + blood pressure

Set 7: + diet + blood pressure

Set 8: + physical activity + diet + blood pressure

Abbreviations: BMI, body mass index; CVH, cardiovascular health; HPFS, Health Professional’s Follow-up Study; LS7, Life’s Simple 7; NHANES: the National Health and Nutrition Examination Survey; NHS, Nurses’ Health Study; NHSII, Nurses’ Health Study II.
